# Advances in smart nanotechnology-supported photodynamic therapy for cancer

**DOI:** 10.1038/s41420-024-02236-4

**Published:** 2024-11-11

**Authors:** Guangyao Li, Cong Wang, Binghui Jin, Tao Sun, Kang Sun, Shuang Wang, Zhe Fan

**Affiliations:** 1 Department of Oncology, Cancer Hospital of Dalian University of Technology, Dalian, China; 2 Department of General Surgery, the Third People’s Hospital of Dalian, Dalian Medical University, Dalian, China; 3 Liaoning Province Key Laboratory of Corneal and Ocular Surface Diseases Research, the Third People’s Hospital of Dalian, Dalian University of Technology, Dalian, China; 4 Department of Digestive Endoscopy, The First Affiliated Hospital of Dalian Medical University, Dalian, China; 5 Department of Endocrinology, The Second Affiliated Hospital of Dalian Medical University, Dalian, China

**Keywords:** Targeted therapies, Drug development

## Abstract

Cancer has emerged as a formidable challenge in the 21st century, impacting society, public health, and the economy. Conventional cancer treatments often exhibit limited efficacy and considerable side effects, particularly in managing the advanced stages of the disease. Photodynamic therapy (PDT), a contemporary non-invasive therapeutic approach, employs photosensitizers (PS) in conjunction with precise light wavelengths to selectively target diseased tissues, inducing the generation of reactive oxygen species and ultimately leading to cancer cell apoptosis. In contrast to conventional therapies, PDT presents a lower incidence of side effects and greater precision in targeting. The integration of intelligent nanotechnology into PDT has markedly improved its effectiveness, as evidenced by the remarkable synergistic antitumor effects observed with the utilization of multifunctional nanoplatforms in conjunction with PDT. This paper provides a concise overview of the principles underlying PS and PDT, while also delving into the utilization of nanomaterial-based PDT in the context of cancer treatment.

## Facts

- Precision and targeting: Photodynamic therapy (PDT) provides high precision in targeting cancer cells, resulting in minimal side effects compared to conventional treatments.
- Role of photosensitizers (PS): Effective PDT depends on photosensitizers that produce reactive oxygen species (ROS) upon light activation, leading to the apoptosis of cancer cells.
- Integration with nanotechnology: Nanomaterial-based PDT improves the delivery, stability, and targeting efficiency of photosensitizers, enhancing overall therapeutic outcomes.
- Challenges with current PS: Most photosensitizers are hydrophobic, which reduces their solubility and efficiency. Nanotechnology provides solutions by enhancing solubility and selective accumulation in target tissues.
- Clinical applications: Advances in nanotechnology have demonstrated promising results in preclinical and clinical settings; however, further research is necessary for widespread clinical adoption.

## Open questions

- Optimizing photosensitizers: How can new generations of photosensitizers be developed to have better water solubility, targeting specificity, and reduced side effects?
- Nanomaterial safety: What are the long-term biocompatibility and toxicity effects of utilizing various nanomaterials in PDT?
- Overcoming biological barriers: What strategies can enhance the selective accumulation of photosensitizers in tumor tissues while minimizing adverse effects on healthy cells?
- Combination therapies: How can PDT be effectively combined with other cancer therapies, such as chemotherapy and immunotherapy, to improve overall treatment efficacy?
- Clinical translation: What are the key challenges in translating nanotechnology-supported PDT from research to routine clinical practice, and how can these challenges be addressed?

## Introduction

Cancer has emerged as a significant social, public health, and economic challenge in the 21st century. In 2022, ~20 million new cancer cases were reported globally, with 9.7 million cancer-related deaths [1]. Traditional cancer treatments, such as surgical resection, radiotherapy, chemotherapy, immunotherapy, and molecular targeted therapy, have shown success in treating early-stage tumors. However, for advanced tumors, these traditional methods often exhibit limited efficacy and can induce severe side effects and treatment resistance [2]. Despite substantial progress in cancer treatment, there is an urgent need for continuous innovation to develop more effective therapeutic approaches.

Photodynamic therapy (PDT) is a contemporary, non-invasive treatment that involves the local or systemic application of photosensitizers (PS), which accumulate in pathological tissues, to treat both non-tumorous diseases and various cancers [3]. PDT can be applied before or after chemotherapy, radiotherapy, or surgery without compromising these treatments. One of the primary mechanisms of PDT involves the production of reactive oxygen species (ROS) via PS. This therapeutic strategy leverages the accumulation of PS in tumor tissues, which, upon activation by a specific wavelength of light, releases ROS, including singlet oxygen, superoxide anions, and hydroxyl radicals, resulting in cancer cell death [4]. Notably, PDT produces cytotoxic ROS only when the PS is activated; without external light activation, the toxicity of PS remains minimal [5]. Furthermore, compared to traditional cancer treatments, PDT has fewer side effects and higher targeting specificity, as it treats only the cells or tissues exposed to light [6].

Despite the numerous advantages of PDT in cancer treatment, its clinical application faces challenges due to the properties of PS. Most PS drugs are hydrophobic, resulting in low solubility in water and a tendency to aggregate under physiological conditions, thereby reducing the efficiency of ROS production [7]. Even when some PS drugs are modified to enhance water solubility, their selective accumulation in target tissues or cells remains insufficient for successful clinical application [8]. Therefore, developing effective delivery systems or new PS to overcome biological barriers in PS delivery is crucial for advancing PDT.

Nanomaterials have made significant progress in drug delivery, disease diagnosis, and treatment, playing an essential role in PDT [9]. As carriers or active agents, they enhance the stability and targeting ability of PS and co-deliver other anticancer drugs to achieve combined cancer therapy [10]. The hydrophilicity of nanomaterials increases the solubility of PS in water, thereby enhancing its cellular uptake. Using nanoparticles, PS can be passively targeted to tumors through the enhanced permeability and retention (EPR) effect [11]. Furthermore, surface-modified nanoparticles can improve the cellular specificity of PS and reduce adverse effects on surrounding healthy tissues [12]. These advancements provide crucial support for further optimizing and clinically applying PDT. This review focuses on PDT supported by smart nanotechnology and its applications in cancer treatment.

## The principle of photodynamic therapy

PDT is a non-invasive cancer treatment that utilizes ROS generated by PS. PDT involves three essential components: PS, a light source, and oxygen. Individually, these components are non-toxic; however, their combined action triggers a photochemical reaction that generates highly reactive singlet oxygen. Singlet oxygen rapidly induces cytotoxic effects in cells, leading to apoptosis or necrosis.

**Fig. 1:**
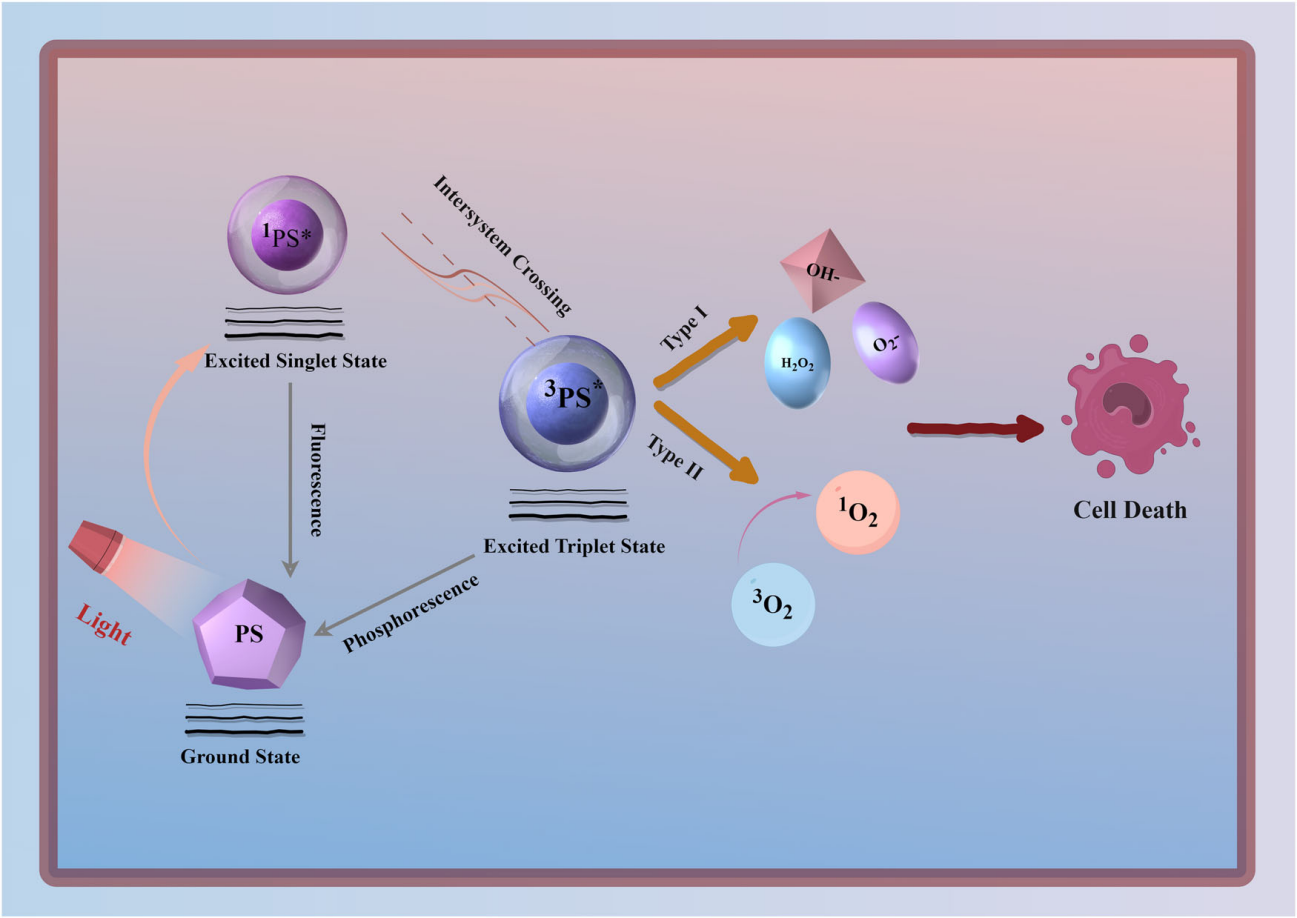
The principle of PDT. By Figdraw.

Upon exposure to a specific wavelength of light, PS transitions from the ground state to an unstable excited singlet state, then undergoes intersystem crossing to an excited triplet state. Excited singlet state PS can return to the ground state by releasing fluorescence or thermal energy. Similarly, excited triplet state PS can return to the ground state by releasing phosphorescence or thermal energy [13]. More importantly, excited triplet state PS interacts with surrounding molecules to produce ROS via Type I and Type II reactions [14]. In Type I reactions, excited triplet state PS directly interacts with cellular substrates, generating radicals through hydrogen atom or electron transfer, which subsequently react with oxygen to produce toxic ROS. Type II reactions involve energy transfer between excited PS and ground state molecular oxygen (^3^O_2_), resulting in the formation of singlet oxygen, a highly reactive form of ROS (Fig. 1). Mechanistically, generating ROS through Type II reactions is generally simpler than through Type I reactions, and most PS are believed to act primarily via Type II rather than Type I mechanisms. The reactive species formed during PDT ultimately cause irreversible damage to target tissues and cells. Therefore, PS that efficiently generates oxidative products and singlet oxygen are crucial for the effectiveness of PDT.

The antitumor effects of PDT involve three primary mechanisms [15–17]. First, ROS triggers oxidative stress responses, activating protein kinase pathways, transcription factors, and cytokine expression, and increasing levels of apoptosis-related factors, leading to cell apoptosis and necrosis. Second, ROS damages tumor vascular endothelial cells, causing vascular dysfunction and thrombosis, which significantly reduces the nutrient and oxygen supply to tumor cells. Third, ROS-induced apoptosis and necrosis further stimulate the release of pro-inflammatory factors and the activation of T-cells, triggering an acute inflammatory response and eliciting a systemic anticancer immune response. These interrelated mechanisms collectively enhance PDT’s overall inhibitory effect on tumors. Additionally, the destruction of tumor cells results in the production of new tumor antigens and increased expression of stress proteins. Tumor cell fragments killed by PDT are phagocytosed by macrophages, leading to acute inflammation, leukocyte infiltration, and dendritic cell activation [18].

## Photosensitizers

Besides the light source and oxygen, PS are indispensable in the PDT process. PS are substances that absorb specific wavelengths of light and initiate photochemical or photophysical reactions [19]. The mechanisms by which PDT induces tumor cell death largely depend on the type and dosage of PS, as well as the characteristics of the target cells [13].

PS must have certain characteristics to ensure their effectiveness and safety in PDT. First, PS must exhibit high tumor selectivity in vivo, preferentially accumulating in tumor tissues and remaining inactive in the absence of light [20]. Second, the photosensitizing effect of PS should only be activated by specific wavelengths of light, requiring an absorption peak typically between 600 and 800 nm [21]. Additionally, PS must have a high singlet oxygen quantum yield to ensure sufficient ROS production upon light exposure [22]. Chemically, PS should be of high purity, stable at room temperature, and easily soluble in body tissues to ensure even distribution and efficacy. They should avoid overlap with the absorption bands of other substances to prevent unnecessary phototoxicity [23]. Finally, the synthesis process of PS should be cost-effective, simple, and easily scalable to promote widespread clinical application [24]. Considering these characteristics collectively helps ensure the effectiveness and safety of PS in PDT. In order to understand each photosensitizer in more detail, the following table lists specific photosensitizers and their associated information (Table 1).

**Table 1:** Photosensitizers and their application and properties in photodynamic therapy.

Class	Photosensitizer	Wavelength (nm)	Approval Status	Applications	Features	References
Porphyrin	Porfimer sodium	630	FDA approved	Esophageal cancer, lung cancer, endobronchial cancer, biliary cancer, bladder cancer, brain cancer, gastric cancer	Strong near-infrared light absorption, definite efficacy, complex composition, and slow metabolism, may cause erythema and constipation	[136–138]
Porphyrin precursor	5-Aminolevulinic acid	635	FDA approved	Basal cell carcinoma, squamous cell carcinoma, skin cancer, bladder cancer, esophageal cancer	May cause mild to moderate photokeratosis	[139, 140]
Chlorin	Temoporfin (mTHPC)	652	EU approved	Pancreatic cancer, biliary cancer, breast cancer metastases	Good deep penetration and tumor selectivity, but may cause phototoxicity and poor water solubility, laser equipment dependent, photosensitivity also needs attention	[141, 142]
	Talaporfin (NPe6)	660	Japan approved	Liver cancer, colon cancer, brain cancer	Poor solubility	[137]
	Photochlor (HPPH)	655	USA approved	BCC, lung, head and neck cancer	Weak fluorescence signal	[143]
Bacteriochlorin	Padoporfin (TOOKAD)	762	EU approved	Prostate cancer	Strong light penetration, effective in hypoxic environments, low photosensitivity side effects	[144]
Texaphyrin	Motexafin lutetium (Lu-Tex)	732	In clinical trials	Breast cancer	Good photosensitivity, good tissue selectivity, photosensitivity toxic side effects	[145]
Phthalocyanine (Pc)	AlPcSn	675	In clinical trials	Age-related macular degeneration, prostate cancer	High photothermal stability, strong coordination ability, poor water solubility, easy aggregation	[146, 147]
	Silicon phthalocyanine (PC4)	675	In clinical trials	Cutaneous T-cell lymphoma		[148]
Cyanine	Indocyanine green (ICG)	805	FDA approved (Diagnosis of fluorescence-guided cancer surgery)	Topical melanoma PDT	Photosensitizer easy to aggregate, strong near-infrared light absorption, fast metabolism with uncertain mechanism of action, low oxygen production	[149]

### First-generation photosensitizers

The earliest discovery of hematoporphyrin as a PS is attributed to Oscar Raab, a medical student in Munich. In 1904, Professor Von Tappeiner described this phenomenon as “photodynamic action,” laying the foundation for PDT research [25]. In the 1970s, Thomas Dougherty and his team were the first to introduce PS into commercial-scale therapy, developing hematoporphyrin derivative (HpD) from a mixture of water-soluble porphyrins. The first clinically used PS for cancer treatment was HpD, later purified and named Photofrin [26]. As the first clinically approved PS, Photofrin has been widely used to treat various cancers, including non-small cell lung cancer, bladder cancer, skin cancer, esophageal cancer, and brain cancer [27, 28]. HpD demonstrates greater tumor tissue selectivity and lower photosensitivity in the skin.

However, first-generation PS have some limitations [29, 30]. Their absorption wavelengths are relatively short, limiting their penetration into deep tissues. Additionally, due to lower chemical purity and a long half-life, patients may experience photosensitivity for weeks after PDT. Although first-generation PS achieved some clinical success, these limitations have driven researchers to explore new generations of PS.

### Second-generation photosensitizers

Since the 1980s, scientists have been researching second-generation PS [31]. Research on second-generation PS has seen continuous development and effort. Medicinal chemists have extensively worked to discover more effective PS. They have proposed hundreds of compounds as potential candidates for anticancer PDT. Although some compounds have entered clinical trials, the number of PS formally approved for anticancer PDT remains limited. 5-Aminolevulinic acid (ALA) is a precursor in heme synthesis and is not inherently photosensitive. When exogenous 5-ALA is introduced into the body, it selectively accumulates in rapidly proliferating cells and is enzymatically converted into protoporphyrin IX, becoming an active PS [32]. This characteristic makes 5-ALA a prodrug in PDT, requiring conversion to protoporphyrin IX within the body for effectiveness. This discovery has enabled the use of ALA or its derivatives topically or orally in various clinical applications [33].

Second-generation PS have notable features compared to first-generation PS [34, 35]. First, they exhibit superior chemical purity and singlet oxygen generation rates, making them more effective in PDT. Additionally, their peak absorption within the 650–800 nm range allows better penetration into deeper tissues, providing a feasible approach for treating deep-seated tumors. Moreover, second-generation PS have fewer side effects, higher selectivity for cancer tissues, and can be cleared from the body more quickly than first-generation PS.

However, second-generation PS have some limitations [36]. Their poor water solubility can lead to aggregation under physiological conditions, reducing ROS yield in PDT. Furthermore, their hydrophobicity limits their use for intravenous injection [37]. Successful clinical application of PS requires a balance between hydrophilicity and lipophilicity, necessitating new drug delivery methods to overcome this limitation [29].

### Third-generation photosensitizers

Despite the improved therapeutic efficacy of second-generation PS, the complex tumor microenvironment and glutathione consumption of ROS reduce PDT’s toxic efficiency [38]. Moreover, the hydrophilicity, tumor selectivity, and in vivo clearance rate of second-generation PS are suboptimal. Therefore, further research to optimize PS is imperative.

Current research focuses on developing third-generation PS to enhance tumor specificity and reduce the impact on healthy tissues [29]. These improvements are primarily achieved by modifying existing PS for tumor-specific targeting or encapsulating them in efficient delivery carriers [39]. Third-generation PS are designed to more selectively bind to tumor cells or the tumor microenvironment, promoting tumor localization and reducing damage to surrounding healthy tissues.

## Nanomaterials for photodynamic therapy

### The versatility of nanotechnology in photodynamic therapy

In PDT, nanotechnology overcomes the limitations of traditional PS and enhances therapeutic efficacy. Nanotechnology has significantly advanced medicine, particularly in precision and targeted therapy. Nanomaterials possess unique physicochemical properties, including a large surface area-to-volume ratio and tunable surface functionalization, making them excellent drug carriers [40]. These properties enhance the solubility, stability, and bioavailability of drugs, enabling precise control over drug release. Nanocarriers can effectively improve the water solubility and biocompatibility of PS. The strong hydrophobicity of most PS limits their applications in vivo. Nanomaterials can bind to PS via covalent or non-covalent interactions, maintaining their dispersion and stability in aqueous environments, thereby preserving their photoactivity [41]. For example, binding with polymer nanoparticles allows PS to be effectively delivered to tumor tissues, reducing degradation before reaching the target [42].

Nanotechnology offers the potential for targeted delivery of PS. Modifying the surface of nanoparticles enhances the affinity of PS for tumor cells, improving selectivity and treatment efficiency [43]. This targeting strategy extends beyond PS, delivering other drugs and therapeutic agents, enhancing efficacy while reducing toxicity to normal tissues [44]. The multifunctionality of nanomaterials enables the integration of multiple therapeutic modalities on a single platform. Besides delivering PS, nanoparticles can be used for diagnostics (e.g., integrating contrast agents or fluorescent markers) or other therapies [45]. This combinational therapy strategy targets multiple pathways in tumors simultaneously, increasing treatment comprehensiveness and effectiveness.

**Fig. 2:**
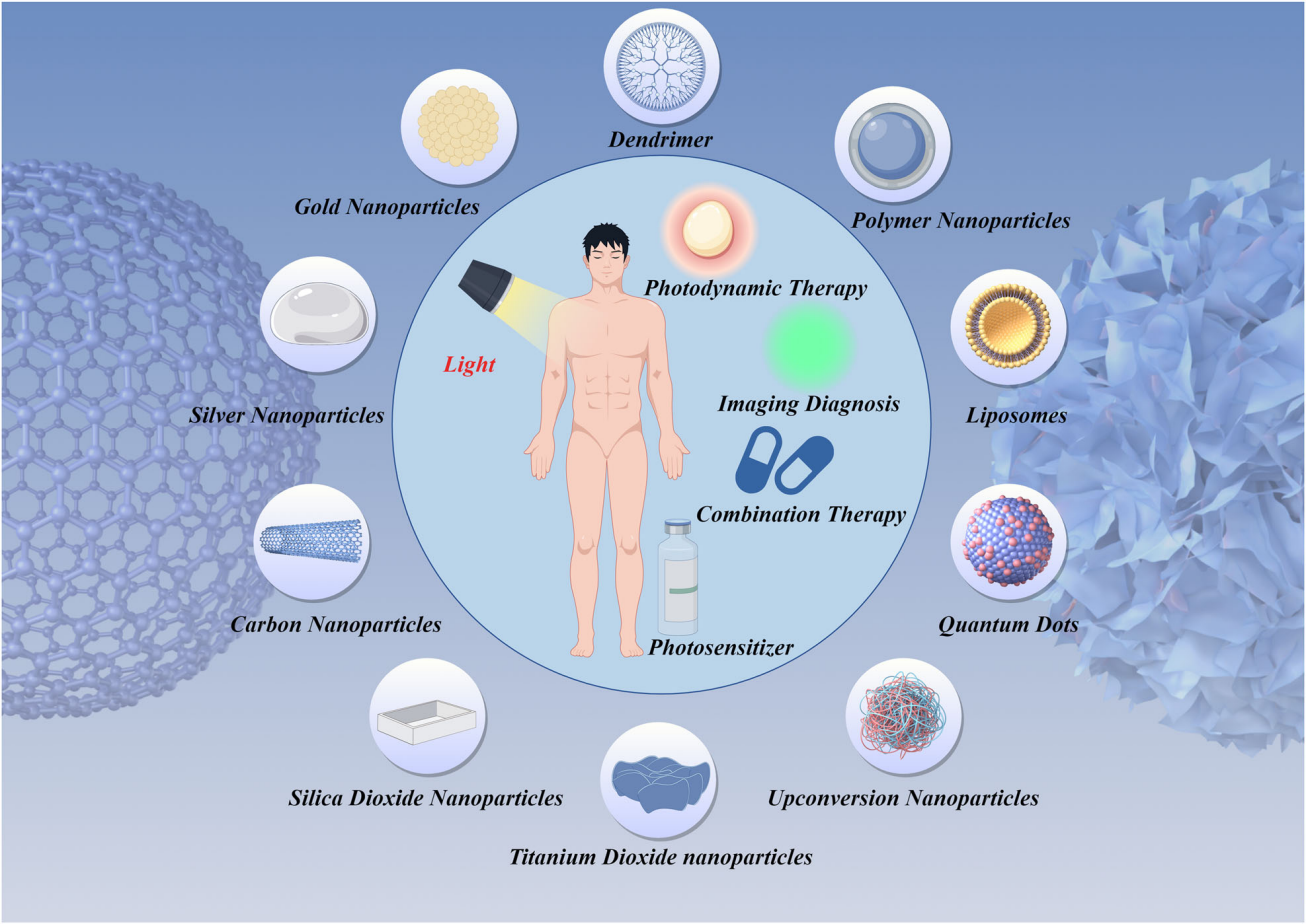
Some inorganic and organic nanomaterials. By Figdraw.

Nanotechnology also addresses challenging issues like tumor multidrug resistance and microenvironment-induced treatment limitations. Nanocarriers enhance PDT efficacy by modulating the tumor microenvironment, such as improving local oxygen supply or regulating the extracellular matrix [46]. Below, we present several inorganic and organic nanomaterials (Fig. 2).

### Inorganic nanomaterials

#### Gold nanoparticles

Gold nanoparticles (AuNPs) are widely used in drug delivery and cancer therapy due to their high surface area, low toxicity, and excellent biocompatibility [47]. Through gold-thiol chemistry, AuNPs are easily surface-functionalized and suitable for binding various drug molecules and ligands [48]. AuNPs can be synthesized into various shapes, such as nanospheres, nanorods, and nanoshells, which exhibit strong absorption in the near-infrared region, making them suitable for photothermal therapy (PTT) and PDT [49]. Additionally, AuNPs are used to deliver PS passively and actively to target areas, enhancing drug delivery efficiency. AuNPs can carry and deliver hydrophobic PS to tumor tissues via the EPR effect [50]. Due to their high biocompatibility and ease of surface functionalization, AuNPs have garnered significant attention in diagnostics and bioimaging [51].

Although AuNPs may aggregate and be cleared by the immune system in physiological media, this can be prevented by using biocompatible materials [52]. Additionally, the surface plasmon resonance peak of AuNPs needs to be tuned to avoid interfering with the phototherapeutic efficacy of PS [53]. Uniform morphology is crucial for ensuring consistent optical effects and therapeutic functions.

Advances in nanotechnology have enabled researchers to synthesize AuNPs with ideal properties for PDT applications. In summary, AuNPs have become important tools in drug delivery, diagnostics, and cancer therapy due to their unique physicochemical properties and ease of surface modification. With appropriate optimization, AuNPs hold great potential for clinical therapy.

#### Silver nanoparticles

Silver is widely recognized as a potent natural antibiotic owing to its robust antimicrobial properties, and has been extensively employed in eradicating diverse microorganisms. In recent years, Silver nanoparticles (AgNPs) have emerged as pivotal agents in antimicrobial therapy owing to their large surface area, minimal dosage requirements, and notable antibacterial effects [54].

Another noteworthy aspect of AgNPs is their potential application in PDT. Studies have demonstrated that AgNPs can produce singlet oxygen, which is essential for PDT. Sun and his colleagues effectively attained a synergistic effect by integrating silver with PDT through the utilization of polyethyleneimine modified with Chlorin e6 (Ce6) as the ligand for AgNPs [55]. The surface plasmon resonance (SPR) effect of silver enhances the production of singlet oxygen during PDT, while these ROS further promote the oxidative dissolution of silver ions, leading to antibacterial effects. Additionally, AgNPs can induce apoptosis in tumor cells by amplifying oxidative stress at the tumor site and can be further optimized in PDT through rational design and surface modification [56].

Owing to their effective antibacterial properties and potential in PDT, AgNPs have garnered significant attention in research and application. Through the optimization of morphology and excitation conditions, AgNPs can play a pivotal role in cancer treatment, providing novel therapeutic approaches and strategies.

#### Carbon nanomaterials

Carbon nanomaterials are well-suited for cancer PDT owing to their outstanding optical properties, mechanical strength, good biocompatibility, low toxicity, EPR effect, and capability to generate ROS. Among them, carbon nanotubes (CNTs), fullerenes, and graphene-based nanomaterials are the most commonly utilized.

CNTs are exceptional carbon nanomaterials, typically classified into multi-walled carbon nanotubes (MWCNTs) and single-walled carbon nanotubes (SWCNTs) [57]. They are regarded as one-dimensional structures derived from rolled graphene and are highly esteemed in drug delivery because of their distinctive structure and properties. They play a crucial role in drug delivery due to their properties, such as high drug-loading efficiency and excellent transmembrane permeability [58]. Furthermore, CNTs can undergo chemical modification for functionalization, thereby enhancing their performance in PDT [59]. SWCNTs have garnered particular attention for their potential application in cancer treatment as efficient carriers for hydrophobic PS [60]. Despite the considerable potential of CNTs in PDT, their cytotoxicity to normal tissues severely hampers their practical medical application [61].

Fullerenes are all-carbon materials comprising pure carbon atoms with distinctive three-dimensional structures, such as spherical, ellipsoidal, cylindrical, or tubular forms, with the C60 form being the most extensively researched [62]. Fullerenes can generate ROS and superoxide anions upon light exposure, making them effective PS in PDT [63]. Nevertheless, the inadequate water solubility and tendency to form nanoclusters of unmodified fullerenes restrict their PDT applications, and their intrinsic toxicity poses a challenge [64]. Consequently, researchers have devised various strategies for surface modification or functionalization to enhance their water solubility, augment PDT efficacy, and broaden their potential for combination therapy [65]. Moreover, fullerene derivatives have been formulated for targeted drug delivery systems, specifically targeting nuclear pore complexes and tumor vasculature [66]. These functionalized fullerenes, with their high resistance to photobleaching, photochemical reactivity, and capacity to self-assemble into supramolecular structures, are deemed superior PS.

Graphene-based nanomaterials, comprising graphene quantum dots (GQDs) and graphene oxide (GO), find extensive application in cancer therapy owing to their expansive surface area, exceptional thermal and optical characteristics, and favorable biocompatibility, particularly in anticancer drug delivery and PDT [67].

GQDs, zero-dimensional graphene nanomaterials, have garnered attention for their potential to co-deliver PS and anticancer drugs in combined PDT and chemotherapy. Studies have shown that GQDs exhibit excellent performance in PDT, with outstanding singlet oxygen quantum yield [68]. Nevertheless, despite their advantages in PDT, GQDs also present potential toxicity concerns, including DNA damage and selective inhibition of antioxidant enzyme activity [69]. Research has found that fluorine modification can enhance the biocompatibility of GQDs [70].

GO, endowed with excellent water solubility and diverse chemical functionalities, surpasses graphene as a PS delivery carrier [71]. The oxygen groups on GO’s surface facilitate the modification with hydrophilic macromolecules, targeting agents, and active agents, extending its biological applications and reducing toxicity [72]. Furthermore, the remarkable fluorescence quenching capability of GO enhances its utility in PDT [73]. Besides serving as a PS carrier, GO itself also possesses PTT and PDT effects, and its combined therapy can effectively prevent tumor recurrence and metastasis [74].

#### Silicon dioxide nanoparticles

Silicon dioxide (SiO_2_) forms a tetrahedral structure consisting of one silicon atom and four oxygen atoms. SiO_2_ nanoparticles are classified by pore size into microporous (<2 nm), mesoporous (2-50 nm), and macroporous (>50 nm) types [75]. Although SiO_2_ is inactive in PDT, it is often used to encapsulate PS in PDT due to its non-toxicity, chemical inertness, and optical transparency [76]. Additionally, the hydroxyl groups on the SiO_2_ surface facilitate chemical functionalization, enhancing its application in drug delivery research [77].

Mesoporous silica nanoparticles (MSNs) are considered ideal PS carriers due to their suitable pore structure, tunable pore size, and large surface area [78]. MSNs, with their high loading capacity, multifunctional surface chemistry, and good biocompatibility, are key nanodelivery systems for PDT and combination therapies, ensuring stable loading and precise release of PS [79].

#### Titanium dioxide nanoparticles

Titanium dioxide (TiO_2_) exhibits photoactivity under visible or ultraviolet (UV) light and demonstrates high stability, low dark toxicity, and excellent biocompatibility, making it useful for anticancer PDT applications [80]. However, pure TiO_2_ can only generate ROS under UV light activation, and the poor tissue penetration capability of UV light limits its application in PDT. Additionally, UV light exposure can activate proteins, hemoglobin, and melanin, potentially posing health risks [81]. Therefore, TiO_2_ must be functionalized to broaden its application range in PDT.

Through functionalization, TiO_2_ can serve as a PS to generate singlet oxygen and is noted for its tunable band gaps and positions, excellent photostability, low toxicity, high catalytic activity, abundance, and cost-effectiveness [82]. Functionalized TiO_2_ nanoparticles can be used alone or as composite materials with other compounds, effectively applied in PDT for treating malignant tumors or inactivating antibiotic-resistant bacteria [81]. These characteristics make TiO_2_ highly promising for PDT and other medical applications.

#### Upconversion nanoparticles

Upconversion nanoparticles (UCNPs) convert low-energy near-infrared light into high-energy UV light via a nonlinear anti-Stokes mechanism, making them promising carriers for PDT [83]. During PDT, photons emitted by UCNPs activate PS, resulting in the generation of ROS. Compared to two-photon PDT, UCNPs offer higher conversion efficiency and require a lower power density. Additionally, the emission wavelengths of UCNPs match the absorption spectra of PS, enhancing the efficiency of ROS production [84]. Moreover, UCNPs have additional advantages, including low toxicity, narrow emission bandwidth, long decay time, resistance to photobleaching, and absence of autofluorescence background. These advantages enable UCNPs to effectively address the issue of light penetration depth in PDT [85]. Concurrently, the next generation of PS, termed PUNP-type PS, consists of photosensitive compounds and nanoparticles. Their core can convert photon energy into higher energy and absorb infrared radiation, allowing deeper tissue penetration [86]. Antibody labeling allows these nanoparticles to selectively accumulate in tumor tissues, demonstrating significant potential for applications.

#### Quantum dots

Quantum dots (QDs), unique zero-dimensional nanomaterials, possess distinctive optical and optoelectronic properties due to quantum confinement effects, making them highly suitable for PDT [87]. QDs exhibit high emission quantum yields, tunable optical properties, and facile surface modification, enabling the adjustment of emission wavelengths to meet the absorption requirements of various PS [88]. Their broad absorption spectrum and narrow emission range enhance imaging and therapeutic accuracy. Additionally, QDs can serve as donors in fluorescence resonance energy transfer (FRET) studies of biomolecular dynamics [89].

In PDT, QDs function as both diagnostic tools and therapeutic agents. T Their emission properties can be conjugated with cancer-targeting molecules, such as antibodies or peptides, to precisely locate tumors [90]. Through FRET or upconversion mechanisms, QDs can activate nearby PS, producing therapeutic effects upon light exposure [91]. This enables QDs to function effectively under near-infrared light, enhancing tissue penetration depth and the therapeutic efficacy of PS.

Despite the numerous advantages of QDs, their application in PDT faces several challenges. Firstly, toxic heavy metals like cadmium in QDs pose risks to biological systems, making the development of cadmium-free QDs a crucial research direction [92]. Secondly, QDs exhibit low drug and PS loading efficiencies, necessitating new optimization strategies [93]. Lastly, hydrophobic QDs have poor solubility in aqueous solutions, necessitating improved techniques to enhance their applicability in biological systems [94].

### Organic nanomaterials

#### Liposomes

Liposomes are biocompatible nanovesicles composed of phosphatidylcholine and cholesterol, extensively used for drug and PS delivery [95]. Liposomes can encapsulate both hydrophilic and hydrophobic substances and achieve passive tumor targeting through the EPR effect, making them highly promising for PDT applications.

In PDT, liposomes encapsulate PS and reach tumor tissues via the EPR effect. Under specific near-infrared irradiation, PS is activated to generate ROS, leading to liposome rupture due to the action of ROS or intracellular phospholipase, thereby releasing PS to act on tumor tissues [96]. This activatable liposome design effectively enhances passive tumor targeting while reducing damage to surrounding normal tissues by PS.

Experimental and clinical studies have demonstrated the significant efficacy of liposomes as PS delivery vehicles in targeting various cancer cells and inhibiting tumor angiogenesis [95]. For instance, liposome-based PS delivery systems have demonstrated significant therapeutic effects against metastatic melanoma cells, breast cancer cells, skin cancer cells, and tumor vascular endothelial cells.

#### Polymeric nanoparticles

Polymeric nanoparticles (PNPs) are a rapidly developing material in the biomedical field. Their excellent biocompatibility, controllable size, and surface functionalization properties confer great potential for PNPs in drug delivery, gene therapy, and cancer treatment [97]. Meanwhile, combining PDT with PNPs can significantly enhance therapeutic efficacy and reduce side effects [98].

PNPs are typically prepared by methods such as emulsification, solvent evaporation, and nanoprecipitation, with most of these materials being non-toxic and biodegradable [99]. Their size can be precisely controlled by adjusting preparation conditions, thereby enhancing the biodistribution and permeability of drugs. Furthermore, the surface of PNPs can be chemically modified to achieve targeted delivery. Encapsulating PS within PNPs can prevent early degradation and enhance accumulation in tumor tissues [100]. Such modifications also enable targeted delivery of PS, reducing damage to normal tissues.

#### Dendritic polymers

Dendritic polymers are nano-sized branched polymers, serving as ideal delivery vehicles for PS in PDT due to their highly branched structure and monodispersity [101]. These carriers possess strong permeability, efficient drug release, high solubility, and high loading capacity, along with various advantages such as colloidal stability, biological stability, and storage stability. Notably, dendritic polymers have been applied for the delivery of 5-ALA as a PS precursor [102]. Drugs can be encapsulated in the core or conjugated on the surface of dendritic polymers, making them attractive carriers for anticancer therapy. The broad synthesis and application of dendritic polymers offer new avenues and broad prospects, particularly in the medical field and PDT [103].

These characteristics render dendritic polymers effective delivery systems in PDT, protecting drugs from initial degradation, enhancing drug penetration into target tissues, and reducing systemic toxicity, thereby improving treatment efficacy and safety.

## Nanomaterial-based photodynamic therapy

**Fig. 3:**
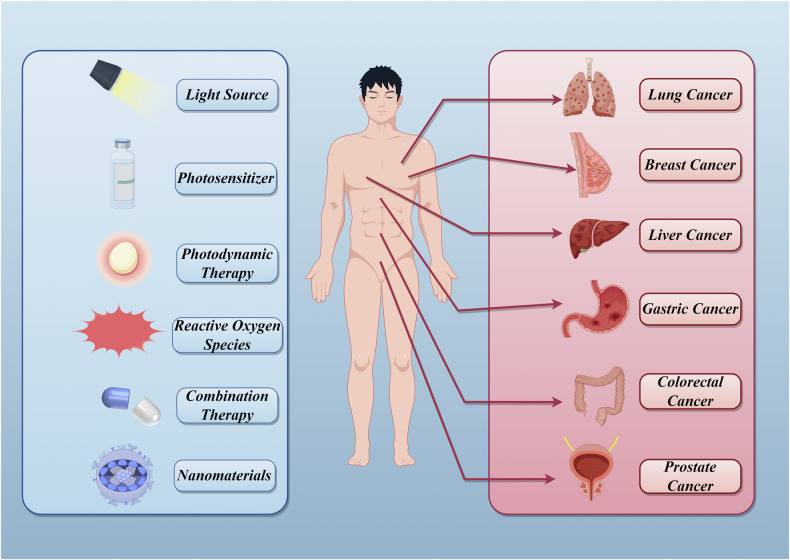
Application of nanomaterials-based PDT in the top six cancers in terms of incidence in GLOBOCAN 2022 statistics. By Figdraw.

Traditional cancer treatments, such as surgery, radiotherapy, and chemotherapy, although often effective, have significant limitations. Some cancers do not respond effectively to these traditional methods due to treatment resistance, metastasis, and patient relapse, making cancer treatment more complex and challenging [104]. To address these challenges, nanoparticle-based PDT is emerging as a promising alternative. Utilizing the unique properties of nanomaterials, PDT can achieve more precise tumor targeting, enhance the stability and efficacy of PS, significantly improve treatment outcomes, and reduce side effects on healthy tissues. Next, we will discuss the application of nanoparticle-based PDT in the six most prevalent cancers, according to GLOBOCAN 2022 statistics (Fig. 3).

### Lung cancer

In 2022, lung cancer was the leading cause of cancer incidence and mortality worldwide, with nearly 2.5 million new cases and over 1.8 million deaths, accounting for 12.4% of all diagnosed cancers and 18.7% of all cancer deaths globally [1]. In response, researchers have conducted numerous studies focused on lung cancer treatment.

Taşkonak et al. [105] developed hypericin (HY)-loaded chitosan nanoparticles (HY-CH-NP) to enhance HY intracellular uptake and prolong its action time, thereby improving its PDT efficacy. Experimental results showed that after PDT treatment, HY-CH-NP significantly reduced the viability of A549 human lung cancer cells to 56%, increased ROS production by 1.6 times, and led to a cell death rate of 40%. Additionally, increased lactate dehydrogenase release indicated enhanced necrosis.

Güleryüz et al. [106] encapsulated PS IR780 and the anticancer agent curcumin (Cur) in polyethylene glycol (PEG)-terminated MSNs to create the nanocarrier Cur&IR780@MSN. Experimental results demonstrated that Cur&IR780@MSN exhibited a synergistic therapeutic effect, reducing lung cancer cell viability by up to 78%, showcasing exceptional performance.

Liao et al. [107] used supramolecular self-assembly to load the theophylline derivative d17 and PS Ce6 into nanoparticles, forming d17-Ce6 NPs. These nanoparticles exhibited good hydrophilicity and stability in non-small cell lung cancer tissues, showing enhanced retention and accumulation in tumor sites. Experimental results indicated that d17-Ce6 NPs could synergistically inhibit tumor growth without significant toxicity.

Zhao et al. [108] designed a nanoparticle called the porphyrin-cholesterol conjugate (TPPC) to address lung cancer through combined PDT and immunotherapy. TPPC accumulated at tumor sites, induced immunogenic cell death, and promoted immune responses. This combined treatment strategy significantly inhibited tumor growth and eliminated metastasis, providing a theoretical basis for the potential use of PDT with immune checkpoint inhibitors in lung cancer treatment.

Crous et al. [109] constructed a nanobioconjugate (NBC) by combining AuNPs and aluminum phthalocyanine chloride (AlPcS4Cl) with antibodies to enhance PDT efficacy against lung cancer stem cells. Results showed that NBC successfully conjugated and, compared to AlPcS4Cl alone, exhibited significant cytotoxicity and promoted cell death, thereby enhancing PDT efficacy and effectively targeting lung cancer stem cells.

### Breast cancer

According to 2022 data, female breast cancer is the second most common cancer globally, with approximately 2.3 million new cases, representing 11.6% of all cancer cases [1]. Additionally, it is the fourth leading cause of cancer-related deaths worldwide, resulting in 666,000 deaths, accounting for 6.9% of all cancer deaths.

Montaseri et al. [110] developed spherical Janus nanoparticles (JNP) consisting of gold and polyacrylic acid (Au/PAA). These nanoparticles were selectively functionalized on one side with folic acid (FA) and thiol PEG amine (SH-PEG-NH2) to ensure biocompatibility and targeting, while the other side was loaded with 5-ALA for PDT. Results demonstrated that the functionalized FA-Au/PAA-ALA JNP exhibited high cytotoxicity against MCF-7 breast cancer cells in in vitro PDT experiments, indicating potential applications in tumor therapy.

Thomas-Moore et al. [111] investigated glycoconjugated gold nanoparticles (gluAuNP) for targeted PDT. They used polyacrylamide (PAA)-glycans to assess glycan binding to breast cancer cell lines SK-BR-3 and MDA-MD-231, finding that galactose derivative PAA was preferentially taken up by breast cancer cells. Subsequently, they modified AuNPs with galactose derivative ligands and attached amine derivatives of Ce6 to the nanoparticle surface via amide bonds. Results indicated that these dual-functionalized nanoparticles could effectively target and kill breast cancer cells, demonstrating the potential for targeted PDT applications.

Aljarrah et al. [112] utilized silica nanoparticles as carriers for the PS safranin (SF) in PDT against MCF-7 breast cancer cells. The study evaluated the cytotoxicity of encapsulated and bare SF at various concentrations, determining the optimal concentration and exposure time for eradicating MCF-7 cells under red laser irradiation. Results demonstrated that encapsulated SF improved efficacy by 50% in concentration and 78% in exposure time compared to bare SF, significantly enhancing PDT effects. This suggests that silica nanoparticles can enhance the bioavailability of SF in target cells, thereby increasing PDT efficacy, and demonstrating the potential of silica nanoparticles as a PDT drug delivery system.

Wu et al. [113] developed a multifunctional biomimetic nanoplatform, named 4T1Mem@PGA-Ce6/Ola (MPCO), for the co-delivery of Ce6 and Olaparib (Ola), aimed at overcoming the limitations of PDT caused by tumor cell repair mechanisms and low immune responses. This nanoplatform achieved efficient tumor targeting and cellular internalization through cell membrane camouflaging, resulting in significant synergistic antitumor effects under laser irradiation. Additionally, MPCO activated the cGAS-STING pathway, generating cytokines and inducing systemic antitumor immune responses by promoting immunogenic cell death and dendritic cell maturation. This study demonstrated the potential of nanoplatforms that combine PDT, chemotherapy, and immunotherapy in antitumor treatments.

Li et al. [114] proposed a novel small molecule self-assembled nanoprodrug for PDT targeting triple-negative breast cancer (TNBC). Researchers co-delivered an activatable PS and a ROS-resistant inhibitor (SSZ) linked by disulfide bonds to TNBC cells. The nanoprodrug was decomposed by overexpressed glutathione in TNBC cells, releasing the ROS-resistant inhibitor and activating the PS, enabling PDT and fluorescence imaging. Results showed that the nanoprodrug effectively inhibited tumor growth and distant metastasis in both subcutaneous and orthotopic TNBC mouse models while minimizing off-target toxicity in normal tissues. This strategy offers a new approach for PDT treatment and is expected to improve TNBC treatment outcomes.

### Colorectal cancer

In 2022, there were over 1.9 million new cases of colorectal cancer globally, resulting in ~904,000 deaths, accounting for nearly 10% of all new cancer cases and deaths. Overall, colorectal cancer ranks third in incidence and second in mortality worldwide [1].

Simelane et al. [115] synthesized and characterized a novel aluminum phthalocyanine derivative, AlClPcTS41, to explore its effectiveness as a PS. The study combined AlClPcTS41 with polyethylene glycol-coated copper-gold bimetallic nanoparticles (PEG-CuAuNPs), forming AlClPcTS41-PEG-CuAuNPs. The presence of amide bonds was confirmed by FTIR, and their crystal structure, morphology, and size were analyzed using XRD, TEM, and DLS. Experimental results demonstrated that both AlClPcTS41 and AlClPcTS41-PEG-CuAuNPs exhibited significant PDT effects in vitro on Caco-2 cells, markedly reducing tumor cell numbers and potentially enhancing PDT-induced cytotoxicity by triggering apoptotic pathways.

Jahedi et al. [116] combined cerium dioxide (CeNPs)-coated calcium carbonate nanoparticles (CaNPs) with the anticancer drug 5-fluorouracil (5-FU) and functionalized these nanoparticles with FA to develop a novel drug-loaded nanocomposite, Ca.FU.Ce.FA NCs. These composites were further combined with GO and modified with AgNPs to form an oxygen-generating drug delivery system, FU.FA@NS. The modified mesenchymal stem cells (MSCs) successfully loaded and retained these therapeutics with minimal impact on their functionality. Upon UVA irradiation, FU.FA@NS.MSCs co-cultured with CT26 cells exhibited increased tumor cell apoptosis via ROS-mediated mitochondrial pathways.

Zhang et al. [117] developed a nanoparticle named Fe_3_O_4_@BSA-CE6, consisting of a Fe_3_O_4_ core and a bovine serum albumin (BSA) shell modified with Ce6. This nanoparticle was used to combine ferroptosis and PDT for colorectal cancer treatment. Fe_3_O_4_@BSA-CE6 nanoparticles can release ferrous ions and Ce6 in the acidic tumor microenvironment. Under laser irradiation, Ce6 generates ROS, inducing apoptosis. More importantly, the presence of ferrous ions promotes ROS and lipid overload production, thereby inducing ferroptosis through lipid peroxidation. Additionally, due to the magnetic properties of Fe_3_O_4_, these nanoparticles exhibit excellent T2-weighted MRI characteristics. With its synergistic induction of apoptosis and ferroptosis, superior imaging capabilities, and component safety, this nanoparticle shows great potential for future clinical applications.

De Almeida Junior et al. [118] developed gold shell-isolated nanorods combined with toluidine blue O (TBO), termed AuSHINRs@TBO, demonstrating enhanced phototoxic effects on human colorectal tumor cells (Caco-2). AuSHINRs@TBO further reduced Caco-2 cell viability through oxidative processes, including hydrogen peroxide production. Langmuir monolayer and PM-IRRAS analyses revealed electrostatic interactions between AuSHINRs@TBO and negatively charged groups in Caco-2 lipid extracts, leading to ROS generation and oxidative reactions that ultimately triggered cell death.

Khaled et al. [119] loaded Foslip onto silica nanoparticles. They then functionalized the nanoparticle surface to target carcinoembryonic antigen (CEA), enabling targeted fluorescence imaging and PDT for colorectal cancer. The results showed that these anti-CEA targeted nanoparticles significantly enhanced CEA-specific fluorescence signals in vitro and markedly increased colorectal cancer cell death after light irradiation. In vivo experiments demonstrated a substantial reduction in tumor volume following treatment. This study suggests that anti-CEA functionalized Foslip nanoparticles have potential clinical applications in targeted fluorescence imaging and PDT for colorectal cancer.

### Prostate cancer

Prostate cancer ranks as the second most common cancer worldwide and is particularly prevalent among men. Its incidence escalates with age, notably among individuals aged over 60. In 2022, an estimated 1.5 million new cases of prostate cancer were diagnosed globally, resulting in ~397,000 deaths [1].

Aydingogan et al. [120] synthesized a novel hybrid nanoparticle, the luminescent superparamagnetic Ag2S- Fe_3_O_4_ (AS-SPION), through ligand exchange. They then loaded the nanoparticle with 5-ALA to facilitate combined PDT and PTT for prostate cancer cells. These nanoparticles are engineered to deliver dual-modal imaging and enhanced phototherapeutic effects. Initially, the research team assessed the photothermal potential of these nanoparticles in solution and established the optimal therapeutic parameters. They then confirmed the efficacy of the combined therapy in experimental studies with prostate cancer cell lines, examining mechanisms including ROS generation, apoptosis/necrosis, and live/dead imaging. This study underscored the benefits of hybrid nanoparticles in offering dual-modal imaging and improved phototherapy for prostate cancer, while also delineating the differences between combined therapy and monotherapy.

Zhang et al. [121] designed an intelligent nanocarrier RB-M, which loads rose bengal (RB) for PS delivery, overcoming some limitations of PDT. A light-emitting diode (LED) activation device enabled the programmed release of RB, and its subsequent diffusion into the cytoplasm-activated PDT. Experimental results showed that prostate cancer cells treated with RB-M exhibited good PDT effects under continuous “405–580 nm” irradiation, achieving similar results in both 2D cell cultures and 3D cancer cell spheroids. This intelligent nanocarrier design holds promise in overcoming light penetration limitations in PDT, offering a new treatment option for cancer patients unsuitable for chemotherapy.

Güleryüz et al. [122] developed an innovative PDT strategy aimed at overcoming the limitations of conventional PDT in targeting deep-seated cancer cells. They engineered a nanoplateform, MC540/ZnPc-UCNP@Au, which combines near-infrared light activation with dual photosensitizer encapsulation, thereby enhancing the therapeutic effect on PC3 prostate cancer cells. This nanoplateform integrates multiple functionalities, including UCNP synthesis, porous silica coating, and Au functionalization, all aimed at enhancing PDT efficacy. Experimental findings demonstrated that the MC540/ZnPc-UCNP@Au nanoplateform produced a significant amount of ROS under near-infrared light excitation, confirming its effective photodynamic impact on prostate cancer cells. Additionally, the platform’s capability for visualizable internalization and adsorption provides potential for in situ cellular imaging diagnostics.

Mesquita et al. [123] synthesized three innovative fluorinated porphyrin derivatives and encapsulated them in polyvinylpyrrolidone (PVP), enhancing their solubility and optical properties. In vitro experiments revealed that these PVP formulations (PS1@PVP, PS2@PVP, and PS3@PVP) exhibited negligible dark cytotoxicity at tested concentrations. However, PS2@PVP and PS3@PVP demonstrated significant photodynamic effects on PC3 prostate cancer cells under red light irradiation. Further cellular localization and protein analysis indicated that PS2@PVP and PS3@PVP predominantly localized in mitochondria, reduced the expression of the anti-apoptotic protein Bcl-2, and activated caspase-dependent apoptotic pathways.

Ghosh et al. [124] engineered a dual-mode nanocarrier utilizing upconversion nanoparticles (UCNPs) for synergistic chemotherapy and PDT. They encapsulated the chemotherapy drug doxorubicin (DOX) and tetracarboxyphthalocyanine zinc within a metal-organic framework (ZIF-8), and surface-coated the nanocarrier with amine-PEG for controlled drug release. Additionally, they employed the prostate cancer-specific ligand FA to enhance targeting efficiency. Experimental results revealed that PSMA-positive cells (LNCaP) exhibited enhanced uptake of the FA-conjugated nanocomposites in comparison to PSMA-negative cells (DU145). Under acidic conditions, the PEG coating suppressed DOX release; however, upon near-infrared irradiation, both ROS production and DOX release were facilitated. Cytotoxicity experiments demonstrated that near-infrared irradiation markedly diminished LNCaP cell viability, underscoring the effectiveness of this near-infrared targeted PDT system in augmenting synergistic therapeutic outcomes.

### Gastric cancer

In recent decades, the incidence of gastric cancer has shown a steady decline, largely attributed to advancements in lifestyle and medical care. Nonetheless, recent studies have reported a rising incidence of gastric cancer among younger populations. In 2022, the global incidence of gastric cancer surpassed 968,000 new cases, resulting in close to 660,000 deaths, underscoring the continued significance of gastric cancer as a major global health concern [1].

Guo et al. [125] devised a pioneering drug delivery system, PTX@GO-PEG-OSA, which integrates paclitaxel (PTX) into polyethylene glycol (PEG)-modified and oxidized sodium alginate (OSA)-functionalized GO nanosheets. This drug delivery system demonstrates pH and temperature-sensitive drug release characteristics. In comparison to free PTX, PTX@GO-PEG-OSA exhibits augmented antitumor efficacy in the treatment of gastric cancer. Upon near-infrared irradiation, PTX@GO-PEG-OSA produces excessive ROS, which targets mitochondrial respiratory chain complexes, leading to decreased ATP production and effective suppression of P-glycoprotein efflux pump function. This mechanism markedly enhances the therapeutic efficacy of PTX@GO-PEG-OSA against drug-resistant gastric cancer cells, with minimal observable toxicity. PTX@GO-PEG-OSA, integrating chemotherapy, PTT, and PDT, represents a promising approach for overcoming PTX resistance.

Situ et al. [126] devised a targeted drug delivery platform, ACSN/Fe3O4@MSNs-iRGD, incorporating novel ACSN and the active targeting peptide iRGD to augment the efficacy of PDT. Experimental findings demonstrated the favorable active targeting capability of this drug towards gastric cancer cells, facilitating efficient mitochondrial penetration. The PS released by ACSN significantly suppressed mitochondrial aerobic respiration, preserving oxygen and inducing substantial ROS production. Moreover, the Fe3O4 core-shell structure offered exceptional T2 dark contrast, facilitating real-time tumor monitoring via magnetic resonance imaging. Integrating PDT and MRI imaging functionalities, this drug platform presents a comprehensive and efficacious approach to tumor diagnosis and treatment, with the potential to surmount the constraints of conventional cancer therapies.

Chen et al. [127] formulated a novel ternary copper-based chalcogenide nanoplateform, CuS-NiS2, characterized by outstanding photothermal and photodynamic capabilities. Upon 808 nm near-infrared irradiation, CuS-NiS2 induces the generation of ROS, triggers the Bcl-2/Bax pathway activation, leading to apoptosis in human gastric cancer cells. Furthermore, the co-administration of CuS-NiS2 with an 808 nm near-infrared laser induces necroptosis through modulation of the MLKL/CAPG pathway. Magnetic resonance imaging findings demonstrated the remarkable contrast enhancement effects of CuS-NiS2.

Ding et al. [128] devised a novel diagnostic system, Cy1395-NPs, aimed at augmenting the efficacy of PDT in gastric cancer treatment. This system relies on thio-substituted PS, employing a sulfur substitution strategy to diminish the S1-T1 energy gap and facilitate intersystem crossing, thereby enhancing the efficiency of ROS generation. Upon laser irradiation, Cy1395-NPs exhibited stable spectral properties, excellent biocompatibility, and a high yield of singlet oxygen. Cellular experiments demonstrated the specific targeting of MKN45 cells by Cy1395-NPs through integrin αvβ3-mediated cRGD endocytosis, resulting in mitochondrial accumulation and phototoxicity with minimal dark toxicity. In vivo experiments revealed the selective accumulation of Cy1395-NPs at the tumor site and pronounced inhibition of tumor growth under laser irradiation, inducing apoptosis in tumor cells.

Li et al. [129] formulated a novel nanoparticle termed IRCB@M, which enhances PDT through dual mechanisms. These nanoparticles self-assembled to incorporate the photosensitizer IR780 and the glutaminase inhibitor CB-839, and were enveloped with cancer cell membranes for homologous targeting. Inhibiting glutamine metabolism and aerobic respiration decreased the levels of NADPH and reduced glutathione, alleviating hypoxia and augmenting the PDT effect mediated by IR780. Both in vitro and in vivo experiments demonstrated the significant enhancement of the antitumor effect on gastric cancer by these nanoparticles, offering a novel strategy to enhance PDT and other ROS-dependent therapies.

### Liver cancer

In 2022, liver cancer caused over 750,000 deaths worldwide, making it the third leading cause of cancer-related deaths and the sixth most common cancer, with approximately 865,000 new cases [1]. The primary types are hepatocellular carcinoma (accounting for 75–85%) and intrahepatic cholangiocarcinoma (accounting for 10–15%). Chronic HBV or HCV infection accounts for 21–55% of hepatocellular carcinoma cases globally. Other risk factors include aflatoxin exposure, heavy alcohol consumption, overweight, type 2 diabetes, and smoking [130].

Guo et al. [131] combined aromatic short peptide Fmoc-L3-OMe with porphyrin derivative m-THPP to co-assemble into supramolecular nanoparticles to enhance antitumor therapeutic effects. Morphological analysis of HepG2 cells revealed photoreactive behavior and autophagy upon 633 nm laser irradiation. Further non-destructive AFM cellular mechanics studies observed a doubling of HepG2 cell membrane stiffness.

Lu et al. [132] developed a novel gallium-based liquid metal composite nanosphere LM@ZrO2-MN-RGD (LZMR). Experimental results showed that LZMR could generate heat and ROS under near-infrared light and X-ray irradiation, respectively, for PTT and PDT. Additionally, LZMR could deplete glutathione in the tumor microenvironment, enhancing sensitivity to X-rays, ultimately achieving up to 97% tumor inhibition in animal models.

Zeng et al. [133] developed a novel biomimetic oxygen delivery system named BLICP@O2 to improve the efficacy of PDT in hepatocellular carcinoma treatment. This system utilizes a mixture of tumor cell membranes and thermosensitive liposomes to carry oxygen, integrating NIR-II dye and PS. Under specific wavelength irradiation, BLICP@O2 exhibited significant photothermal and photodynamic responses, triggering oxygen release through photothermal action, thereby enhancing photodynamic effects. In vitro and in vivo experiments confirmed that this strategy effectively enhanced PDT efficacy by alleviating tumor hypoxia.

Gao et al. [134] developed a nanoplateform named Ce6@PMTKP for combined chemotherapy and PDT to enhance the efficacy of immune checkpoint blockade therapy against hepatocellular carcinoma. Ce6@PMTKP includes Ce6 for PDT and ROS-sensitive paclitaxel (PTX) for chemotherapy, effectively accumulating at the tumor site and triggering drug release upon light irradiation. Compared to chemotherapy or PDT alone, Ce6@PMTKP combined therapy more effectively eradicated tumors, induced immunogenic cell death, and increased T lymphocyte infiltration, alleviating tumor microenvironment immunosuppression. When combined with anti-PD-L1 immunotherapy, this platform completely ablated primary tumors and significantly inhibited the growth of metastatic tumors.

Our previous study [135] synthesized two halogenated BODIPY compounds, BDPBr2 and BDPCl2, and prepared self-assembled nanoparticles LBPNPs (LBBr2 NPs and LBCl2 NPs) with lenvatinib, coated with Pluronic F127. These nanoparticles were effectively taken up by liver cancer cells and released the drug in acidic environments, enhancing the targeting and antitumor effects of chemotherapy drugs. Through pH-responsive chemo/photodynamic synergistic therapy, LBPNPs more effectively inhibited tumor growth compared to the drugs alone and promoted the caspase cascade reaction.

### Other types of cancer

In the previous sections of this paper, we have explored in detail the application of nanotechnology-supported photodynamic therapy for cancer and its effectiveness in lung, breast, colorectal, prostate, gastric, and liver cancers. However, photodynamic therapy, as an emerging therapeutic tool, has a much wider range of applications. In order to demonstrate the potential of nanotechnology-supported photodynamic therapy in cancer treatment more comprehensively, we have also collected and organized applications in a variety of other cancer types. These applications are intended to provide readers with a comprehensive view of the current status of PDT applications in cancer treatment. In the next section, we will present these applications in a comprehensive manner through a detailed table, including the therapeutic effects and potential value of PDT in several of the major cancer types mentioned in the previous section, as well as in a variety of other cancer types, so that readers can have a more intuitive and comprehensive understanding of the latest progress and prospects of the application of PDT in the field of cancer therapy (Table 2).

**Table 2:** Summary of nanodelivery systems for cancer photodynamic therapy.

Nanodelivery system	Photosensitizer	Cancer type	Therapeutic effect	Reference
HY-CH-NPs	Hypericin	Lung cancer	Significantly reduced cell viability; increased ROS production in the 600 nm group; induced apoptosis and secondary necrosis; increased lactate dehydrogenase release and increased necrosis.	[105]
Cur&IR780@MSN	IR780	Lung cancer	Significant synergistic therapeutic effect; decreased cell viability; increased ROS production; inhibition of cell migration; good biocompatibility; ph-dependent release of curcumin.	[106]
d17-Ce6 NPs	Ce6	Non-small cell lung cancer	It has good hydrophilicity and stability; effectively accumulates in tumor tissues, synergistically inhibits tumor growth without obvious toxicity; effectively absorbed by tumor cells and exerts antitumor effects.	[107]
TPPC NPs	Porphyrin-cholesterol conjugate	Lung cancer	Effectively accumulates at the tumor site, promotes cancer cell death, inhibits metastasis and invasion; induces immunogenic death, increases antitumor response; combined with immunotherapy, enhances antitumor effects.	[108]
AuNP-Ab-AlPcS4Cl	AlPcS4Cl	Lung cancer	Significantly enhanced cytotoxicity and death in the treated group; enhanced PDT effect and destruction of lung cancer stem cells; improved drug transport, increased cytotoxicity, apoptosis, and decreased proliferation and viability of lung cancer stem cells.	[109]
FA-Au/PAA-ALA JNPs	5-ALA	Breast cancer	Good biocompatibility and active targeting; high cancer cell killing efficiency; no significant in vitro dark toxicity.	[110]
GlycoAu NPs	Ce6	Breast cancer	Selectively binds to cancer cells and induces breast cancer cell death; control cells are unaffected.	[111]
Si NPs	Safranin	Breast cancer	Encapsulated Safranin is more potent, generates higher ROS and enhances PDT efficacy.	[112]
4T1Mem@PGA-Ce6/Ola	Ce6/Ola	Breast cancer	Efficient targeting of tumors; Ce6+Ola synergistic antitumor; activation of cGAS-STING pathway and induction of immune response; inhibition of breast cancer growth and prevention of metastatic recurrence.	[113]
SSZ-SS-PS NPs	-	Triple-negative breast cancer	GSH-responsive release; increased ROS; in vivo imaging and antitumor; inhibition of distant metastasis and reduced toxicity.	[114]
AlClPcTS41-PEG-CuAuNPs	AlClPcTS41	Colon cancer	Significant generation of ROS; reduced cell viability; altered cell morphology; increased number of cell deaths; low cell dark toxicity and good biocompatibility.	[115]
FU.FA@NS.MSCs	Ag NP	Colon cancer	Increases apoptosis of tumor cells via ROS-mediated mitochondrial pathway, enhances cellular uptake efficiency and intrinsic toxicity, disrupts intracellular oxidative status, alters mitochondrial membrane integrity and induces apoptosis in a caspase-dependent manner.	[116]
Fe3O4@BSA-CE6 NPs	CE6	Colorectal cancer	Generation of ROS under laser irradiation to induce apoptosis; generation of lipid peroxides catalyzed by ferrous ions to induce iron death; T2-weighted magnetic resonance imaging capability; in vivo experiments in mice showed basically normal organ morphology and good safety.	[117]
AuSHINRs@TBO	TBO	Colorectal cancer	Significantly reduced cell survival; photothermal effects combined with oxidative processes enhanced ROS generation and induced cell death.	[118]
Anti-CEA-targeted silica NPs	Foslip	Colorectal cancer	Light irradiation significantly led to cell death and tumor size reduction after PDT treatment.	[119]
Ag2S-Fe3O4 (AS-SPION)	5-ALA	Prostate cancer	Significantly increased cell mortality through combination therapy, with optimal treatment parameters identified in LnCap, PC3, and Du145 cell lines after optimization of PTT conditions, demonstrating a bimodal, enhanced phototherapeutic effect that yielded significant differences compared to monotherapy.	[120]
RB-M	RB	Prostate cancer	Exhibited good PDT effect on both two-dimensional 22RV1 cells and three-dimensional cancer cell spheroids, successfully controlling the proliferation of cell spheroids.	[121]
MC540/ZnPc-UCNP@Au	MC540/ZnPc	Prostate cancer	A large amount of ROS was generated, leading to a significant reduction in PC3 cell viability; meanwhile, the nanoplatform was easily internalized/adsorbed by the cells, which has the potential for in situ cellular imaging diagnostics.	[122]
PVP NPs	Fluorinated porphyrin derivatives	Prostate cancer	Activation of apoptosis is triggered through the mitochondrial pathway, which regulates apoptosis-related proteins, and the caspase-dependent pathway is involved in the apoptotic response.	[123]
UCNP@MOF-DOX-TCZnPc@PEG/FA	TCZnPc	Prostate cancer	Release of DOX and TCZnPc, enhanced release by low pH degradation of MOF; Successful uptake by LNCaP cells, decreased cell viability, good therapeutic efficacy; Effective killing of cancer cells by the dual-mode system.	[124]
PTX@GO-PEG-OSA NSs	-	Gastric cancer	It produces more obvious antitumor effects on gastric cancer, effectively inhibits the exocytosis pump function of P-gp, and achieves better therapeutic effects on PTX-resistant GC without obvious toxicity.	[125]
ACSN/Fe_3_O_4_@MSNs-iRGD	ACSN	Gastric cancer	Good active targeting ability, effective penetration into mitochondria, saving oxygen and generating large amounts of ROS under laser excitation; real-time monitoring of tumors by MRI imaging, significant suppression of tumor volume.	[126]
CuS-NiS_2_ NPs	-	Gastric cancer	Induces ROS generation, leads to apoptosis of human gastric cancer cells through the Bcl-2/Bax pathway, and triggers necrotic apoptosis through the MLKL/CAPG pathway. Effectively inhibited human gastric cancer in vivo and in vitro without significant side effects.	[127]
Cy1395-NPs	Reactive thiosensitizers	Gastric cancer	It actively targets tumors and exerts mitochondrial selectivity, exhibiting good fluorescence imaging; it showed good inhibitory effects on gastric cancer cells in both in vivo and ex vivo experiments, leading to significant tumor suppression and cell apoptosis.	[128]
IRCB@M NPs	IR780	Gastric cancer	By blocking the Gln metabolic pathway and aerobic respiration, it reduces NADPH and GSH levels and exerts a dual amplification effect on IR780-mediated lethal PDT, significantly limiting tumor progression with low biological cytotoxicity and high biocompatibility.	[129]
Self-assembled peptide NPs	m-THPP	Liver cancer	Significant changes in the hardness of HepG2 cell membranes suggest the possibility of reducing the malignancy of hepatocellular carcinoma by modulating the hardness of cancer cells and are promising for use in tracking tumor tissue in vivo and killing tumors via PDT.	[131]
LM@ZrO_2_-MN-RGD (LZMR)	-	Liver cancer	LZMR produces thermal effects and abundant ROS, while decreasing GSH in the tumor area and enhancing sensitivity to X-rays and RT. In vivo experiments showed significant tumor growth inhibition by the combination treatment.	[132]
BLICP@O_2_	Ce6	Hepatocellular carcinoma	Through precise and controllable oxygen delivery and release, it effectively relieves tumor hypoxia and significantly improves PDT efficacy. Dual laser approach triggers controlled and rapid release of oxygen to improve the hypoxic microenvironment of hepatocellular carcinoma.	[133]
Ce6@PMTKP	Ce6	Hepatocellular carcinoma	Combination therapy is more effective in eliminating tumors, inducing immunogenic death, promoting immune cell maturation and infiltration, and attenuating immunosuppression compared to chemotherapy or photodynamic therapy alone. In combination with anti-PD-L1 therapy, it induces complete tumor regression and inhibits metastasis.	[134]
LBPNPs (LBBr_2_ NPs和LBCl_2_ NPs)	BDPBr_2_和BDPCl_2_	Liver cancer	Significantly inhibits tumor growth, promotes cascade reaction of caspase apoptotic proteases, and enhances targeting of chemotherapeutic agents through pH-responsive chemo/photodynamic synergistic therapy.	[135]
E7-ICG-BSA NPs	ICG	Cervix cancer	With slow-release properties, it can be delivered to the lymph nodes in a targeted manner. Combined with infrared laser irradiation of the photosensitizer to produce a photo-oxidative reaction, it can effectively stimulate the effect of T-cells, thus enhancing the antitumor effect.	[150]
MWCNT-PEI·SAH-FITC-PEG-FA	ICG	Cervix cancer	Enables FL/PA imaging and thermography of tumors, as well as in vitro phototherapy of cancer cells and in vivo phototherapy of xenograft tumor models with good colloidal stability, hemocompatibility, and high affinity for FA receptor overexpression in cancer cells.	[151]
Pluronic® P123 (HYP/P123)	Hypericin (HYP)	Cervix cancer	Highly efficient selective phototoxicity to cervical cancer cells with little damage to normal cells; inhibits cell colony formation, possibly preventing recurrence; and inhibits tumor cell migration and invasion.	[152]
Porphyrin-HDL Nanoparticles (PLP)	Pyropheophorbide-lipid	Thyroid cancer	PLP accumulates preferentially in the tumor and directs PDT to specifically kill tumor cells without affecting normal tissues. Long-term studies have confirmed that PLP-PDT completely ablates tumors and protects the thyroid and nerves.	[153]
Au@TNA@MB NPs	MB	Bladder cancer	Significantly reduces cancer cell survival, potent phototoxicity; slight phototoxicity to normal cells, good therapeutic selectivity and safety.	[154]
PLZ4-Porphyrin nanoparticles (PNP)	Porphyrin	Bladder cancer	PNP specifically identifies cancer cells and enhances photodynamic diagnosis; eliminates tumors in situ; photodynamic, photothermal and chemotherapy synergize to prolong survival and achieve highly effective treatment.	[155]
CAT-Ce6/F-PEI NPs	Ce6	Bladder cancer	NPs penetrate inside the tumor, relieve hypoxia, increase the effect of PDT, destroy the tumor, reduce systemic toxicity, effectively inhibit the tumor, and are safe and non-toxic.	[156]
NP-PPE/Ce6	Ce6	Pancreatic cancer	Enhanced ROS, increased phototoxicity; strong antitumor efficacy; prolonged circulation time, increased accumulation, rapid release, combined with NIR irradiation significant tumor suppression.	[157]
EGaIn NPs	BPD	Pancreatic cancer	Biocompatible, stable, promote uptake, strong targeting, high ROS; near-infrared activation to eliminate cancer cells; in vivo tumor regression, high necrosis rate, good PDT effect.	[158]
MSNPs	ZnPcOBP	Pancreatic cancer	High photosensitivity, selective delivery, enhanced phototoxicity by cetuximab, and cell line-dependent photocide correlate with EGFR expression levels.	[159]
LDH-PdTCPP	PdTCPP	Melanoma	Single-linear oxygen production was detected, in vivo studies showed significant reduction in tumor volume, PDT performance is highly efficient, low cytotoxicity and biologically safe.	[160]
SLN-AlPc	AlPc	Melanoma	Photoactivated SLN-AlPc effectively reduced the viability of B16-F10 cells in in vitro experiments, mainly by inducing apoptosis.	[161]
PLGA Np	PpIX	Melanoma	At specific concentrations, PLGA Np attenuates the dark toxicity of PpIX while maintaining efficient phototoxicity, with good potential for malignant melanoma PDT.	[162]
TiO2-ZnPc NPs	ZnPc	Glioblastoma	Good biocompatibility; slight phototoxicity on photoactivation, presumed to produce charge separation and generate ROS substances, suitable for PDT for glioblastoma.	[163]
SIWV-pSiNP(ICG)	ICG	Glioblastoma	Enhanced photodynamic force and ICG stability under near-infrared light; targeted to tumor cells, non-toxic and laser-triggered ROS; anticancer efficiency and biocompatibility in glioblastoma mice.	[164]
PB@PMO	5-ALA	Glioma	Induced high accumulation of PpIX in glioma cells with significantly elevated photodynamic effects and enhanced U87MG cytotoxicity; ex vivo imaging showed its accumulation in gliomas, synergistically enhanced PDT, and effectively suppressed tumors.	[165]
CS-ALA-shGBAS NPs	5-ALA	Oral squamous cell carcinoma	Stable dispersion, low toxicity, suitable for cell transfection, exhibits highly efficient mitochondrial targeting and killing of OSCC, with more significant effects on cell viability.	[166]
Sor-Ce6 NPs	Ce6	Oral cancer	Stable targeting, low toxicity, and potentiation promote ROS generation and oxygenation, iron death enhances phototoxicity; antitumor superiority over mixtures due to cellular uptake and Fe ion doping; success attributed to targeting and dual mechanism; potential for bimodal imaging-guided PDT.	[167]
GCDs-Ce6/Pt-EGF	Ce6	Esophageal cancer	Specifically targets tumors, lowers GSH, enhances chemotherapy and PDT; excellent ex vivo imaging, high potential for precision therapy; optimal combination therapy, biologically safe.	[168]
BMIOC	IR820	Esophageal cancer	The BMIOC system is initiated by the tumor microenvironment to enable MR imaging, PTT and PDT; nano-enzymes catalyze oxygen production to enhance PDT, combined with PTT to enhance efficacy; specific to tumors and protect normal tissues.	[169]

## Conclusion and future perspectives

PDT, as a cancer treatment method, presents various advantages and limitations. Compared to traditional chemotherapy, PDT offers greater specificity, exhibiting toxicity to tumor cells exclusively under light irradiation, thereby minimizing damage to normal tissues. Additionally, PDT serves as a viable alternative for patients ineligible for surgery and as an adjuvant therapy post-surgery, potentially reducing the risk of tumor recurrence. Combining chemotherapy with PDT may reduce the required dosage of chemotherapeutic drugs, thereby mitigating their side effects.

However, PDT possesses several limitations as well. Firstly, its primary suitability for localized treatment complicates the eradication of distant tumor cells, especially those embedded in deep tissues. Light penetration in tissues faces efficiency limitations, particularly in deep tissues, due to absorption by endogenous biomolecules. Furthermore, current technologies for PDT light sources present challenges in treating extensive or deeply located tumors. Additionally, the majority of clinically approved PS are porphyrin derivatives, characterized by low bioavailability and prolonged elimination half-lives.

In the realm of nanomedicine, although nanotechnology provides benefits like extended circulation time and enhanced targeting capabilities, it also faces significant challenges. Currently, only a limited number of nanomedicines have received clinical approval, with the majority remaining in the research phase. The distribution of nanomedicines within tumors is heterogeneous; effectively, only a small fraction reaches the tumor tissue. Moreover, nanomedicines that target specific molecules on cancer cell surfaces or within the tumor microenvironment have not yet achieved widespread market application. Therefore, despite the new possibilities that nanotechnology introduces to PDT, overcoming the limitations inherent in traditional nanomedicines necessitates further research and innovation.

## References

[1] Bray F, Laversanne M, Sung H, Ferlay J, Siegel RL, Soerjomataram I, et al. Global cancer statistics 2022: GLOBOCAN estimates of incidence and mortality worldwide for 36 cancers in 185 countries. CA Cancer J Clin. 2024;74:229–63. 38572751 10.3322/caac.21834

[2] Kaur R, Bhardwaj A, Gupta S. Cancer treatment therapies: traditional to modern approaches to combat cancers. Mol Biol Rep. 2023;50:9663–76. 37828275 10.1007/s11033-023-08809-3

[3] Li X, Lovell JF, Yoon J, Chen X. Clinical development and potential of photothermal and photodynamic therapies for cancer. Nat Rev Clin Oncol. 2020;17:657–74. 32699309 10.1038/s41571-020-0410-2

[4] Ming L, Cheng K, Chen Y, Yang R, Chen D. Enhancement of tumor lethality of ROS in photodynamic therapy. Cancer Med. 2021;10:257–68. 33141513 10.1002/cam4.3592 PMC7826450

[5] Sai DL, Lee J, Nguyen DL, Kim Y-P. Tailoring photosensitive ROS for advanced photodynamic therapy. Exp Mol Med. 2021;53:495–504. 33833374 10.1038/s12276-021-00599-7 PMC8102594

[6] Gao P, Wang M, Chen Y, Pan W, Zhou P, Wan X, et al. A COF-based nanoplatform for highly efficient cancer diagnosis, photodynamic therapy and prognosis. Chem Sci. 2020;11:6882–8. 33033601 10.1039/d0sc00847h PMC7500084

[7] Wang C, Zhao P, Jiang D, Yang G, Xue Y, Tang Z, et al. In situ catalytic reaction for solving the aggregation of hydrophobic photosensitizers in tumor. ACS Appl Mater interfaces. 2020;12:5624–32. 31918542 10.1021/acsami.9b21589

[8] Li J, Wang A, Zhao L, Dong Q, Wang M, Xu H, et al. Self-assembly of monomeric hydrophobic photosensitizers with short peptides forming photodynamic nanoparticles with real-time tracking property and without the need of release in vivo. ACS Appl Mater interfaces. 2018;10:28420–7. 30067331 10.1021/acsami.8b09933

[9] Nasseri B, Alizadeh E, Bani F, Davaran S, Akbarzadeh A, Rabiee N, et al. Nanomaterials for photothermal and photodynamic cancer therapy. Appl. Phys. Rev. 2022;9:011317 .

[10] Zhang Y, Wang B, Zhao R, Zhang Q, Kong X. Multifunctional nanoparticles as photosensitizer delivery carriers for enhanced photodynamic cancer therapy. Mater Sci Eng C Mater Biol Appl. 2020;115:111099. 32600703 10.1016/j.msec.2020.111099

[11] Cheng Z, Li M, Dey R, Chen Y. Nanomaterials for cancer therapy: current progress and perspectives. J Hematol Oncol. 2021;14:1–27. 34059100 10.1186/s13045-021-01096-0 PMC8165984

[12] Vankayala R, Hwang KC. Near‐infrared‐light‐activatable nanomaterial‐mediated phototheranostic nanomedicines: an emerging paradigm for cancer treatment. Adv Mater. 2018;30:1706320. 10.1002/adma.201706320 29577458

[13] Pham TC, Nguyen V-N, Choi Y, Lee S, Yoon J. Recent strategies to develop innovative photosensitizers for enhanced photodynamic therapy. Chem Rev. 2021;121:13454–619. 34582186 10.1021/acs.chemrev.1c00381

[14] Algorri JF, Ochoa M, Roldan-Varona P, Rodriguez-Cobo L, Lopez-Higuera JM. Photodynamic therapy: a compendium of latest reviews. Cancers. 2021;13:4447. 34503255 10.3390/cancers13174447 PMC8430498

[15] Donohoe C, Senge MO, Arnaut LG, Gomes-da-Silva LC. Cell death in photodynamic therapy: from oxidative stress to anti-tumor immunity. Biochim Biophys Acta Rev Cancer. 2019;1872:188308. 31401103 10.1016/j.bbcan.2019.07.003

[16] Sharma D, Singh S, Kumar P, Jain GK, Aggarwal G, Almalki WH, et al. Mechanisms of photodynamic therapy. In: Kesharwani, P, editor. Nanomaterials for photodynamic therapy. Elsevier; 2023. p. 41–54.

[17] Gunaydin G, Gedik ME, Ayan S. Photodynamic therapy—current limitations and novel approaches. Front Chem. 2021;9:691697. 34178948 10.3389/fchem.2021.691697 PMC8223074

[18] Huang Y, Guan Z, Dai X, Shen Y, Wei Q, Ren L, et al. Engineered macrophages as near-infrared light activated drug vectors for chemo-photodynamic therapy of primary and bone metastatic breast cancer. Nat Commun. 2021;12:4310. 34262026 10.1038/s41467-021-24564-0 PMC8280231

[19] Yao Q, Fan J, Long S, Zhao X, Li H, Du J, et al. The concept and examples of type-III photosensitizers for cancer photodynamic therapy. Chem. 2022;8:197–209.

[20] Chen D, Xu Q, Wang W, Shao J, Huang W, Dong X. Type I photosensitizers revitalizing photodynamic oncotherapy. Small 2021;17:2006742. 10.1002/smll.202006742 34038611

[21] Zheng B, Zhong D, Xie T, Zhou J, Li W, Ilyas A, et al. Near-infrared photosensitization via direct triplet energy transfer from lanthanide nanoparticles. Chem. 2021;7:1615–25.

[22] Wang X, Song Y, Pan G, Han W, Wang B, Cui L, et al. Exploiting radical-pair intersystem crossing for maximizing singlet oxygen quantum yields in pure organic fluorescent photosensitizers. Chem Sci. 2020;11:10921–7. 34094341 10.1039/d0sc03128c PMC8162435

[23] Roque JA, Barrett PC, Cole HD, Lifshits LM, Shi G, Monro S, et al. Breaking the barrier: an osmium photosensitizer with unprecedented hypoxic phototoxicity for real world photodynamic therapy. Chem Sci. 2020;11:9784–806. 33738085 10.1039/d0sc03008b PMC7953430

[24] Prentice C, Martin AE, Morrison J, Smith AD, Zysman-Colman E. Benzophenone as a cheap and effective photosensitizer for the photocatalytic synthesis of dimethyl cubane-1, 4-dicarboxylate. Org Biomol Chem. 2023;21:3307–10. 36815384 10.1039/d3ob00231d

[25] Kolarikova M, Hosikova B, Dilenko H, Barton-Tomankova K, Valkova L, Bajgar R, et al. Photodynamic therapy: innovative approaches for antibacterial and anticancer treatments. Med Res Rev. 2023;43:717–74. 36757198 10.1002/med.21935

[26] Jia J, Wu X, Long G, Yu J, He W, Zhang H, et al. Revolutionizing cancer treatment: nanotechnology-enabled photodynamic therapy and immunotherapy with advanced photosensitizers. Front Immunol. 2023;14:1219785. 37860012 10.3389/fimmu.2023.1219785 PMC10582717

[27] Kessel D. Photodynamic therapy: a brief history. J Clin Med. 2019;8:1581. 31581613 10.3390/jcm8101581 PMC6832404

[28] McFarland SA, Mandel A, Dumoulin-White R, Gasser G. Metal-based photosensitizers for photodynamic therapy: the future of multimodal oncology? Curr Opin Chem Biol. 2020;56:23–7. 31759225 10.1016/j.cbpa.2019.10.004 PMC7237330

[29] Mfouo-Tynga IS, Dias LD, Inada NM, Kurachi C. Features of third generation photosensitizers used in anticancer photodynamic therapy. Photodiagnosis Photodyn Ther. 2021;34:102091. 33453423 10.1016/j.pdpdt.2020.102091

[30] Zhang J, Jiang C, Longo JPF, Azevedo RB, Zhang H, Muehlmann LA. An updated overview on the development of new photosensitizers for anticancer photodynamic therapy. Acta Pharm Sin B. 2018;8:137–46. 29719775 10.1016/j.apsb.2017.09.003 PMC5925394

[31] Tavakkoli Yaraki M, Liu B, Tan YN. Emerging strategies in enhancing singlet oxygen generation of nano-photosensitizers toward advanced phototherapy. Nanomicro Lett. 2022;14:123. 35513555 10.1007/s40820-022-00856-y PMC9072609

[32] Shinoda Y, Kato D, Ando R, Endo H, Takahashi T, Tsuneoka Y, et al. Systematic review and meta-analysis of in vitro anti-human cancer experiments investigating the use of 5-aminolevulinic acid (5-ALA) for photodynamic therapy. Pharmaceuticals. 2021;14:229. 33800109 10.3390/ph14030229 PMC8000125

[33] Alekseeva P, Efendiev K, Shiryaev A, Rusakov M, Simonova M, Samoylova S, et al. Sublingual administration of 5-aminolevulinic acid for laser-induced photodiagnostics and photodynamic therapy of oral cavity and larynx cancers. Photodiagnosis Photodyn Ther. 2021;34:102289. 33839329 10.1016/j.pdpdt.2021.102289

[34] D’Alessandro S, Priefer R. Non-porphyrin dyes used as photosensitizers in photodynamic therapy. J Drug Deliv Sci Technol. 2020;60:101979.

[35] Kwiatkowski S, Knap B, Przystupski D, Saczko J, Kędzierska E, Knap-Czop K, et al. Photodynamic therapy–mechanisms, photosensitizers and combinations. Biomed Pharmacother. 2018;106:1098–107. 30119176 10.1016/j.biopha.2018.07.049

[36] Chen J, Fan T, Xie Z, Zeng Q, Xue P, Zheng T, et al. Advances in nanomaterials for photodynamic therapy applications: status and challenges. Biomaterials. 2020;237:119827. 32036302 10.1016/j.biomaterials.2020.119827

[37] Park W, Cho S, Han J, Shin H, Na K, Lee B, et al. Advanced smart-photosensitizers for more effective cancer treatment. Biomater Sci. 2018;6:79–90. 10.1039/c7bm00872d PMC5736440 29142997

[38] Fan W, Huang P, Chen X. Overcoming the Achilles’ heel of photodynamic therapy. Chem Soc Rev. 2016;45:6488–519. 27722560 10.1039/c6cs00616g

[39] Plekhova N, Shevchenko O, Korshunova O, Stepanyugina A, Tananaev I, Apanasevich V. Development of novel tetrapyrrole structure photosensitizers for cancer photodynamic therapy. Bioengineering. 2022;9:82. 35200435 10.3390/bioengineering9020082 PMC8868602

[40] Büyüktiryaki S, Keçili R, Hussain CM. Functionalized nanomaterials in dispersive solid phase extraction: advances & prospects. Trends Anal Chem. 2020;127:115893.

[41] Zhang S, Malik S, Ali N, Khan A, Bilal M, Rasool K. Covalent and non-covalent functionalized nanomaterials for environmental restoration. Top Curr Chem. 2022;380:44. 10.1007/s41061-022-00397-3 PMC9372017 35951126

[42] Hong EJ, Choi DG, Shim MS. Targeted and effective photodynamic therapy for cancer using functionalized nanomaterials. Acta Pharm Sin B. 2016;6:297–307. 27471670 10.1016/j.apsb.2016.01.007 PMC4951583

[43] Yu X-T, Sui S-Y, He Y-X, Yu C-H, Peng Q. Nanomaterials-based photosensitizers and delivery systems for photodynamic cancer therapy. Biomater Adv. 2022;135:212725. 35929205 10.1016/j.bioadv.2022.212725

[44] Zhao C, Pang X, Yang Z, Wang S, Deng H, Chen X. Nanomaterials targeting tumor associated macrophages for cancer immunotherapy. J Control Release. 2022;341:272–84. 34813877 10.1016/j.jconrel.2021.11.028

[45] Loh KP, Ho D, Chiu GNC, Leong DT, Pastorin G, Chow EKH. Clinical applications of carbon nanomaterials in diagnostics and therapy. Adv Mater. 2018;30:1802368. 10.1002/adma.201802368 30133035

[46] Cheng X, Xu H-D, Ran H-H, Liang G, Wu F-G. Glutathione-depleting nanomedicines for synergistic cancer therapy. ACS Nano. 2021;15:8039–68. 33974797 10.1021/acsnano.1c00498

[47] Gu X, Xu Z, Gu L, Xu H, Han F, Chen B, et al. Preparation and antibacterial properties of gold nanoparticles: a review. Environ Chem Lett. 2021;19:167–87.

[48] Ramsey AV, Bischoff AJ, Francis MB. Enzyme activated gold nanoparticles for versatile site-selective bioconjugation. J Am Chem Soc. 2021;143:7342–50. 33939917 10.1021/jacs.0c11678

[49] Guerrero-Florez V, Mendez-Sanchez SC, Patrón-Soberano OA, Rodríguez-González V, Blach D, Martínez F. Gold nanoparticle-mediated generation of reactive oxygen species during plasmonic photothermal therapy: a comparative study for different particle sizes, shapes, and surface conjugations. J Mater Chem B 2020;8:2862–75. 32186317 10.1039/d0tb00240b

[50] Xu L, Xu M, Sun X, Feliu N, Feng L, Parak WJ, et al. Quantitative comparison of gold nanoparticle delivery via the enhanced permeation and retention (EPR) effect and mesenchymal stem cell (MSC)-based targeting. ACS Nano. 2023;17:2039–52. 36717361 10.1021/acsnano.2c07295

[51] Oliveira BB, Ferreira D, Fernandes AR, Baptista PV. Engineering gold nanoparticles for molecular diagnostics and biosensing. Wiley Interdiscip Rev Nanomed Nanobiotechnol. 2023;15:e1836. 35932114 10.1002/wnan.1836

[52] Fan M, Han Y, Gao S, Yan H, Cao L, Li Z, et al. Ultrasmall gold nanoparticles in cancer diagnosis and therapy. Theranostics. 2020;10:4944. 32308760 10.7150/thno.42471 PMC7163431

[53] Tang Z, Wu J, Yu X, Hong R, Zu X, Lin X, et al. Fabrication of Au nanoparticle arrays on flexible substrate for tunable localized surface plasmon resonance. ACS Appl Mater Interfaces. 2021;13:9281–8. 33587614 10.1021/acsami.0c22785

[54] Bruna T, Maldonado-Bravo F, Jara P, Caro N. Silver nanoparticles and their antibacterial applications. Int J Mol Sci. 2021;22:7202. 34281254 10.3390/ijms22137202 PMC8268496

[55] Sun P, Ye L, Tan X, Peng J, Zhao L, Zhou Y. Silver nanoparticle-assisted photodynamic therapy for biofilm eradication. ACS Appl Nano Mater. 2022;5:8251–9.

[56] Miranda RR, Sampaio I, Zucolotto V. Exploring silver nanoparticles for cancer therapy and diagnosis. Colloids Surf B Biointerfaces. 2022;210:112254. 34896692 10.1016/j.colsurfb.2021.112254

[57] Solorio-Rodriguez SA, Williams A, Poulsen SS, Knudsen KB, Jensen KA, Clausen PA, et al. Single-walled vs. multi-walled carbon nanotubes: influence of physico-chemical properties on toxicogenomics responses in mouse lungs. Nanomaterials. 2023;13:1059. 36985953 10.3390/nano13061059 PMC10057402

[58] Zare H, Ahmadi S, Ghasemi A, Ghanbari M, Rabiee N, Bagherzadeh M, et al. Carbon nanotubes: smart drug/gene delivery carriers. Int J Nanomed. 2021;16:1681–706. 10.2147/IJN.S299448 PMC7936533 33688185

[59] Naief MF, Mohammed SN, Mayouf HJ, Mohammed AM. A review of the role of carbon nanotubes for cancer treatment based on photothermal and photodynamic therapy techniques. J Organometal Chem. 2023;999:122819.

[60] McKernan P, Virani NA, Faria GN, Karch CG, Prada Silvy R, Resasco DE, et al. Targeted single-walled carbon nanotubes for photothermal therapy combined with immune checkpoint inhibition for the treatment of metastatic breast cancer. Nanoscale Res Lett. 2021;16:9. 33411055 10.1186/s11671-020-03459-x PMC7790975

[61] Peng Z, Liu X, Zhang W, Zeng Z, Liu Z, Zhang C, et al. Advances in the application, toxicity and degradation of carbon nanomaterials in environment: a review. Environ Int. 2020;134:105298. 31765863 10.1016/j.envint.2019.105298

[62] Pan Y, Liu X, Zhang W, Liu Z, Zeng G, Shao B, et al. Advances in photocatalysis based on fullerene C60 and its derivatives: properties, mechanism, synthesis, and applications. Appl Catal B Environ. 2020;265:118579.

[63] Gonzalez Lopez EJ, Sarotti AM, Martínez SR, Macor LP, Durantini JE, Renfige M, et al. BOPHY‐Fullerene C60 dyad as a photosensitizer for antimicrobial photodynamic therapy. Chemistry. 2022;28:e202103884. 34878698 10.1002/chem.202103884

[64] Bai X, Dong C, Shao X, Rahman F-U, Hao H, Zhang Y. Research progress of fullerenes and their derivatives in the field of PDT. Eur J Med Chem. 2024;271:116398. 10.1016/j.ejmech.2024.116398 38614061

[65] Serda M, Szewczyk G, Krzysztyńska-Kuleta O, Korzuch J, Dulski M, Musioł R, et al. Developing [60] fullerene nanomaterials for better photodynamic treatment of non-melanoma skin cancers. ACS Biomater Sci Eng. 2020;6:5930–40. 33320587 10.1021/acsbiomaterials.0c00932

[66] Sun Z, Zhou Y, Li L, Zhou C, Jia W, Liu Y, et al. Inhibiting redox-mediated endothelial migration by gadofullerenes for inducing tumor vascular normalization and improving chemotherapy. Sci Bull. 2023;68:1651–61. 10.1016/j.scib.2023.06.031 37453828

[67] Guo S, Song Z, Ji D-K, Reina G, Fauny J-D, Nishina Y, et al. Combined photothermal and photodynamic therapy for cancer treatment using a multifunctional graphene oxide. Pharmaceutics. 2022;14:1365. 35890259 10.3390/pharmaceutics14071365 PMC9318106

[68] Chen J, Wu W, Zhang F, Zhang J, Liu H, Zheng J, et al. Graphene quantum dots in photodynamic therapy. Nanoscale Adv. 2020;2:4961–7. 36132896 10.1039/d0na00631a PMC9419651

[69] Yao Y, Zhang T, Tang M. The DNA damage potential of quantum dots: toxicity, mechanism and challenge. Environ Pollut. 2023;317:120676. 36395913 10.1016/j.envpol.2022.120676

[70] Li Z, Wang D, Xu M, Wang J, Hu X, Anwar S, et al. Fluorine-containing graphene quantum dots with a high singlet oxygen generation applied for photodynamic therapy. J Mater Chem B. 2020;8:2598–606. 32124889 10.1039/c9tb02529d

[71] Tamtaji M, Tyagi A, You CY, Galligan PR, Liu H, Liu Z, et al. Singlet oxygen photosensitization using graphene-based structures and immobilized dyes: a review. ACS Appl Nano Mater. 2021;4:7563–86.

[72] Yu H, He Y, Xiao G, Fan Y, Ma J, Gao Y, et al. The roles of oxygen-containing functional groups in modulating water purification performance of graphene oxide-based membrane. Chem Eng J. 2020;389:124375.

[73] Xiao X, Zhang Y, Zhou L, Li B, Gu L. Photoluminescence and fluorescence quenching of graphene oxide: a review. Nanomaterials. 2022;12:2444. 35889668 10.3390/nano12142444 PMC9319665

[74] Liu J, Yuan X, Deng L, Yin Z, Tian X, Bhattacharyya S, et al. Graphene oxide activated by 980 nm laser for cascading two-photon photodynamic therapy and photothermal therapy against breast cancer. Appl Mater Today. 2020;20:100665.

[75] Afagwu C, Mahmoud M, Alafnan S, Alqubalee A, ElHusseiny A, Patil S. Pore volume characteristics of clay-rich shale: critical insight into the role of clay types, aluminum and silicon concentration. Arab J Sci Eng. 2022;47:12013–29.

[76] Borzęcka W, Pereira PM, Fernandes R, Trindade T, Torres T, Tome JP. Spherical and rod shaped mesoporous silica nanoparticles for cancer-targeted and photosensitizer delivery in photodynamic therapy. J Mater Chem B. 2022;10:3248–59. 35084012 10.1039/d1tb02299g

[77] Barkat A, Beg S, Panda SK, Alharbi KS, Rahman M, Ahmed FJ. Functionalized mesoporous silica nanoparticles in anticancer therapeutics. Semin Cancer Biol. 2021;69:365–75. 10.1016/j.semcancer.2019.08.022 31442571

[78] Huang R, Shen Y-W, Guan Y-Y, Jiang Y-X, Wu Y, Rahman K, et al. Mesoporous silica nanoparticles: facile surface functionalization and versatile biomedical applications in oncology. Acta Biomater. 2020;116:1–15. 32911102 10.1016/j.actbio.2020.09.009

[79] Wang K, Lu J, Li J, Gao Y, Mao Y, Zhao Q, et al. Current trends in smart mesoporous silica-based nanovehicles for photoactivated cancer therapy. J Control Release. 2021;339:445–72. 34637819 10.1016/j.jconrel.2021.10.005

[80] Sargazi S, Simge E, Gelen SS, Rahdar A, Bilal M, Arshad R, et al. Application of titanium dioxide nanoparticles in photothermal and photodynamic therapy of cancer: an updated and comprehensive review. J Drug Deliv Sci Technol. 2022;75:103605.

[81] Ziental D, Czarczynska-Goslinska B, Mlynarczyk DT, Glowacka-Sobotta A, Stanisz B, Goslinski T, et al. Titanium dioxide nanoparticles: prospects and applications in medicine. Nanomaterials. 2020;10:387. 32102185 10.3390/nano10020387 PMC7075317

[82] Shi Z, Zhang K, Zada S, Zhang C, Meng X, Yang Z, et al. Upconversion nanoparticle-induced multimode photodynamic therapy based on a metal–organic framework/titanium dioxide nanocomposite. ACS Appl Mater interfaces. 2020;12:12600–8. 32096623 10.1021/acsami.0c01467

[83] Jin F, Qi J, Liu D, You Y, Shu G, Du Y, et al. Cancer-cell-biomimetic Upconversion nanoparticles combining chemo-photodynamic therapy and CD73 blockade for metastatic triple-negative breast cancer. J Control Release. 2021;337:90–104. 34274385 10.1016/j.jconrel.2021.07.021

[84] Osuchowski M, Osuchowski F, Latos W, Kawczyk-Krupka A. The use of upconversion nanoparticles in prostate cancer photodynamic therapy. Life. 2021;11:360. 33921611 10.3390/life11040360 PMC8073589

[85] Lee SY, Lee R, Kim E, Lee S, Park YI. Near-infrared light-triggered photodynamic therapy and apoptosis using upconversion nanoparticles with dual photosensitizers. Front Bioeng Biotechnol. 2020;8:275. 32373598 10.3389/fbioe.2020.00275 PMC7179334

[86] Ostańska E, Aebisher D, Bartusik-Aebisher D. The potential of photodynamic therapy in current breast cancer treatment methodologies. Biomed Pharmacother. 2021;137:111302. 33517188 10.1016/j.biopha.2021.111302

[87] El-Shabasy RM, Farouk Elsadek M, Mohamed Ahmed B, Fawzy Farahat M, Mosleh KN, Taher MM. Recent developments in carbon quantum dots: properties, fabrication techniques, and bio-applications. Processes. 2021;9:388.

[88] Kovačova M, Špitalska E, Markovic Z, Špitálský Z. Carbon quantum dots as antibacterial photosensitizers and their polymer nanocomposite applications. Part Part Syst Charact. 2020;37:1900348.

[89] Dos Santos MC, Algar WR, Medintz IL, Hildebrandt N. Quantum dots for Förster resonance energy transfer (FRET). Trends Anal Chem. 2020;125:115819.

[90] Kargozar S, Hoseini SJ, Milan PB, Hooshmand S, Kim HW, Mozafari M. Quantum dots: a review from concept to clinic. Biotechnol J. 2020;15:2000117. 10.1002/biot.202000117 32845071

[91] Kaur A, Kaur P, Ahuja S. Förster resonance energy transfer (FRET) and applications thereof. Anal Methods. 2020;12:5532–50. 33210685 10.1039/d0ay01961e

[92] Gidwani B, Sahu V, Shukla SS, Pandey R, Joshi V, Jain VK, et al. Quantum dots: prospectives, toxicity, advances and applications. J Drug Deliv Sci Technol. 2021;61:102308.

[93] Liu Y, Huang H, Cao W, Mao B, Liu Y, Kang Z. Advances in carbon dots: from the perspective of traditional quantum dots. Mater Chem Front. 2020;4:1586–613.

[94] Mohamed WA, Abd El-Gawad H, Mekkey S, Galal H, Handal H, Mousa H, et al. Quantum dots synthetization and future prospect applications. Nanotechnol Rev. 2021;10:1926–40.

[95] Fahmy SA, Azzazy HME-S, Schaefer J. Liposome photosensitizer formulations for effective cancer photodynamic therapy. Pharmaceutics. 2021;13:1345. 34575424 10.3390/pharmaceutics13091345 PMC8470396

[96] Moghassemi S, Dadashzadeh A, Azevedo RB, Feron O, Amorim CA. Photodynamic cancer therapy using liposomes as an advanced vesicular photosensitizer delivery system. J Control Release. 2021;339:75–90. 34562540 10.1016/j.jconrel.2021.09.024

[97] Indoria S, Singh V, Hsieh M-F. Recent advances in theranostic polymeric nanoparticles for cancer treatment: a review. Int J Pharm. 2020;582:119314. 32283197 10.1016/j.ijpharm.2020.119314

[98] Borzęcka W, Domiński A, Kowalczuk M. Recent progress in phthalocyanine-polymeric nanoparticle delivery systems for cancer photodynamic therapy. Nanomaterials. 2021;11:2426. 34578740 10.3390/nano11092426 PMC8469866

[99] Zielińska A, Carreiró F, Oliveira AM, Neves A, Pires B, Venkatesh DN, et al. Polymeric nanoparticles: production, characterization, toxicology and ecotoxicology. Molecules. 2020;25:3731. 32824172 10.3390/molecules25163731 PMC7464532

[100] Rejinold NS, Choi G, Choy J-H. Recent developments on semiconducting polymer nanoparticles as smart photo-therapeutic agents for cancer treatments—A review. Polymers. 2021;13:981. 33806912 10.3390/polym13060981 PMC8004612

[101] Chis AA, Dobrea C, Morgovan C, Arseniu AM, Rus LL, Butuca A, et al. Applications and limitations of dendrimers in biomedicine. Molecules. 2020;25:3982. 32882920 10.3390/molecules25173982 PMC7504821

[102] Kaczorowska A, Malinga-Drozd M, Kałas W, Kopaczyńska M, Wołowiec S, Borowska K. Biotin-containing third generation glucoheptoamidated polyamidoamine dendrimer for 5-aminolevulinic acid delivery system. Int J Mol Sci. 2021;22:1982. 33671436 10.3390/ijms22041982 PMC7922973

[103] Rout SR, Bandaru R, Kenguva G, Hasan N, Alam MS, Shukla R, et al. Dendrimers in photodynamic therapy. In: Kesharwani, P, editor. Nanomaterials for photodynamic therapy. Elsevier; 2023. p. 281–305.

[104] Gavas S, Quazi S, Karpiński TM. Nanoparticles for cancer therapy: current progress and challenges. Nanoscale Res Lett. 2021;16:173. 34866166 10.1186/s11671-021-03628-6 PMC8645667

[105] Taşkonak B, Aylaz G, Andac M, Güven E, Ozkahraman B, Perçin I, et al. Hypericin-loaded chitosan nanoparticles for enhanced photodynamic therapy in A549 lung cancer cells. Bionanoscience. 2023;13:352–64.

[106] Güleryüz B, Işık A, Gülsoy M. Synergistic effect of mesoporous silica nanocarrier-assisted photodynamic therapy and anticancer agent activity on lung cancer cells. Lasers Med Sci. 2024;39:91. 38491201 10.1007/s10103-023-03969-x PMC10942901

[107] Liao Y, Ye J, Xie L, Mao L, Wei J, Liu C, et al. Carrier-free nanodrug for the chemical-photodynamic synergistic treatment of lung cancer. ACS Appl Nano Mater. 2024;7:5628–36.

[108] Zhao M, Hao D, Wu Q, Li Y, Pei Q, Sun T, et al. Porphyrin cholesterol conjugates for enhanced photodynamic immunotherapy toward lung cancer. ACS Appl Mater interfaces. 2023;15:35927–38. 37471051 10.1021/acsami.3c05825

[109] Crous A, Abrahamse H. Effective gold nanoparticle-antibody-mediated drug delivery for photodynamic therapy of lung cancer stem cells. Int J Mol Sci. 2020;21:3742. 32466428 10.3390/ijms21113742 PMC7311980

[110] Montaseri H, Abrahamse H. Targeted photodynamic therapy technique of Janus nanoparticles on breast cancer. Artif Cells Nanomed Biotechnol. 2024;52:270–7. 38696132 10.1080/21691401.2024.2347369

[111] Thomas-Moore BA, Dedola S, Russell DA, Field RA, Marín MJ. Targeted photodynamic therapy for breast cancer: the potential of glyconanoparticles. Nanoscale Adv. 2023;5:6501–13. 38024308 10.1039/d3na00544e PMC10662151

[112] Aljarrah K, Al-Akhras M-AH, Makhadmeh GN, AlZoubi T, Masadeh MM, Mhareb M, et al. Advancing photodynamic therapy efficiency on MCF-7 breast cancer cells through silica nanoparticles-safranin encapsulation: in-vitro evaluation. J Compos Sci. 2023;7:274.

[113] Wu H, Du X, Xu J, Kong X, Li Y, Liu D, et al. Multifunctional biomimetic nanoplatform based on photodynamic therapy and DNA repair intervention for the synergistic treatment of breast cancer. Acta Biomater. 2023;157:551–65. 36513248 10.1016/j.actbio.2022.12.010

[114] Li B, Tian J, Xie X, Zhang F, Wu C, Shan Y, et al. Overcoming ROS resistance of photodynamic therapy with self‐assembled nano‐prodrugs for efficient triple‐negative breast cancer. Adv Funct Mater. 2024;34:2309524.

[115] Simelane NWN, Matlou GG, Abrahamse H. Photodynamic therapy of aluminum phthalocyanine tetra sodium 2-mercaptoacetate linked to pegylated copper–gold bimetallic nanoparticles on colon cancer cells. Int J Mol Sci. 2023;24:1902. 36768224 10.3390/ijms24031902 PMC9915188

[116] Jahedi M, Meshkini A. Tumor tropic delivery of FU. FA@ NSs using mesenchymal stem cells for synergistic chemo-photodynamic therapy of colorectal cancer. Colloids Surf B Biointerfaces. 2023;226:113333. 37141773 10.1016/j.colsurfb.2023.113333

[117] Zhang Z-j, Liu Z-t, Huang Y-p, Nguyen W, Wang Y-x, Cheng L, et al. Magnetic resonance and fluorescence imaging superparamagnetic nanoparticles induce apoptosis and ferroptosis through photodynamic therapy to treat colorectal cancer. Mater Today Phys. 2023;36:101150.

[118] Mendes de Almeida Junior A, Ferreira AS, Camacho SAS, Gontijo Moreira L, de Toledo KA, Oliveira Jr ON, et al. Enhancing phototoxicity in human colorectal tumor cells through nanoarchitectonics for synergistic photothermal and photodynamic therapies. ACS Appl Mater Interfaces. 2024;16:23742–51. 10.1021/acsami.4c02247 38652860

[119] Khaled YS, Khot MI, Aiyappa-Maudsley R, Maisey T, Pramanik A, Tiernan J, et al. Photoactive imaging and therapy for colorectal cancer using a CEA-affimer conjugated Foslip nanoparticle. Nanoscale. 2024;16:7185. 38506227 10.1039/d3nr04118b PMC10993305

[120] Aydindogan E, Irem K, Onbasli K, Acar HY. Imaging guided PTT-PDT combination therapy of prostate cancer utilizing Ag2S-Fe3O4 hybrid nanoparticles and 5-ALA. Photodiagnosis Photodyn Ther. 2023;41:103381.

[121] Sun B, Liu J, Kim HJ, Rahmat JNB, Neoh KG, Zhang Y. Light-responsive smart nanocarriers for wirelessly controlled photodynamic therapy for prostate cancers. Acta Biomater. 2023;171:553–64. 37739246 10.1016/j.actbio.2023.09.031

[122] Güleryüz B, Ünal U, Gülsoy M. Near infrared light activated upconversion nanoparticles (UCNP) based photodynamic therapy of prostate cancers: an in vitro study. Photodiagnosis Photodyn Ther. 2021;36:102616. 34740839 10.1016/j.pdpdt.2021.102616

[123] Mesquita MQ, Ferreira AR, Maria da Graça P, Ribeiro D, Fardilha M, Faustino MA. Photodynamic therapy of prostate cancer using porphyrinic formulations. J Photochem Photobiol B Biol. 2021;223:112301. 10.1016/j.jphotobiol.2021.112301 34492530

[124] Ghosh S, Gul A, Xu P, Lee S, Rafique R, Kim Y, et al. Target delivery of photo-triggered nanocarrier for externally activated chemo-photodynamic therapy of prostate cancer. Mater Today Chem. 2022;23:100688.

[125] Guo W, Chen Z, Feng X, Shen G, Huang H, Liang Y, et al. Graphene oxide (GO)-based nanosheets with combined chemo/photothermal/photodynamic therapy to overcome gastric cancer (GC) paclitaxel resistance by reducing mitochondria-derived adenosine-triphosphate (ATP). J Nanobiotechnol. 2021;19:146. 10.1186/s12951-021-00874-9 PMC8136184 34011375

[126] Situ J, Yang Y, Zhang L, Yan H, Cheng Y. Integration of O 2-economised tumour-targeted photosensitive magnetic nanomaterials in the diagnosis and therapy of gastric cancer. RSC Adv. 2024;14:9920–32. 38528931 10.1039/d4ra00497c PMC10961965

[127] Chen J, Zhang R, Tao C, Huang X, Chen Z, Li X, et al. CuS–NiS2 nanomaterials for MRI guided phototherapy of gastric carcinoma via triggering mitochondria-mediated apoptosis and MLKL/CAPG-mediated necroptosis. Nanotoxicology. 2020;14:774–87. 32401088 10.1080/17435390.2020.1759727

[128] Ding J, Kang X, Feng M, Tan J, Feng Q, Wang X, et al. A novel active mitochondrion-selective fluorescent probe for the NIR fluorescence imaging and targeted photodynamic therapy of gastric cancer. Biomater Sci. 2022;10:4756–63. 35837996 10.1039/d2bm00684g

[129] Li Z, Li X, Lu Y, Zhu X, Zheng W, Chen K, et al. Improved photodynamic therapy based on glutaminase blockage via tumor membrane coated CB‐839/IR‐780 nanoparticles. Small. 2024;20:2305174. 10.1002/smll.202305174 37875654

[130] Ivanov A, Valuev-Elliston V, Tyurina D, Ivanova O, Kochetkov S, Bartosch B, et al. Oxidative stress, a trigger of hepatitis C and B virus-induced liver carcinogenesis. Oncotarget. 2017;8:3895–932. 27965466 10.18632/oncotarget.13904 PMC5354803

[131] Guo X, Liu D, Dong S, Wang Y, Li M. Self-assembled peptide nanoparticles for photodynamic therapy: morphological and mechanical effects on hepatocellular carcinoma cells. Biomed Mater. 2023;18:045026. 10.1088/1748-605X/acddc2 37345306

[132] Lu X, Song Y, Huang Z, Wang J, Gou L, Pu X, et al. Multi-functional EGaIn-ZrO2 composite-spheres with photo-thermal & photodynamic therapies and enhanced radiotherapy and chemodynamic therapy on liver cancer. J Photochem Photobiol A Chem. 2023;443:114837.

[133] Zeng S, Chen J, Gao R, Chen R, Xue Q, Ren Y, et al. NIR‐II photoacoustic imaging‐guided oxygen delivery and controlled release improves photodynamic therapy for hepatocellular carcinoma. Adv Mater. 2024;36:2308780. 10.1002/adma.202308780 37983859

[134] Gao Y, Su Z, Wang C, Xu J, Hu S, Zhang C, et al. Light-triggered polymeric prodrug and nano-assembly for chemo-photodynamic therapy and potentiate immune checkpoint blockade immunotherapy for hepatocellular carcinoma. Mater Des. 2023;225:111457.

[135] Zong J, Peng H, Qing X, Fan Z, Xu W, Du X, et al. Ph-responsive pluronic F127–lenvatinib-encapsulated halogenated boron-dipyrromethene nanoparticles for combined photodynamic therapy and chemotherapy of liver cancer. ACS Omega. 2021;6:12331–42. 34056385 10.1021/acsomega.1c01346 PMC8154152

[136] Bhattacharya D, Mukhopadhyay M, Shivam K, Tripathy S, Patra R, Pramanik A. Recent developments in photodynamic therapy and its application against multidrug resistant cancers. Biomed Mater. 2023;18:062005. 10.1088/1748-605X/ad02d4 37827172

[137] Kim TE, Chang J-E. Recent studies in photodynamic therapy for cancer treatment: from basic research to clinical trials. Pharmaceutics. 2023;15:2257. 37765226 10.3390/pharmaceutics15092257 PMC10535460

[138] Zhang Q, He J, Yu W, Li Y, Liu Z, Zhou B, et al. A promising anticancer drug: a photosensitizer based on the porphyrin skeleton. RSC Med Chem2020;11:427–37. 33479647 10.1039/c9md00558g PMC7460723

[139] Pignatelli P, Umme S, D’Antonio DL, Piattelli A, Curia MC. Reactive oxygen species produced by 5-aminolevulinic acid photodynamic therapy in the treatment of cancer. Int J Mol Sci. 2023;24:8964. 37240309 10.3390/ijms24108964 PMC10219295

[140] Casas A. Clinical uses of 5-aminolaevulinic acid in photodynamic treatment and photodetection of cancer: a review. Cancer Lett. 2020;490:165–73. 32534172 10.1016/j.canlet.2020.06.008

[141] Yakavets I, Millard M, Zorin V, Lassalle H-P, Bezdetnaya L. Current state of the nanoscale delivery systems for temoporfin-based photodynamic therapy: advanced delivery strategies. J Control Release. 2019;304:268–87. 31136810 10.1016/j.jconrel.2019.05.035

[142] Spring BQ, Rizvi I, Xu N, Hasan T. The role of photodynamic therapy in overcoming cancer drug resistance. Photochem Photobiol Sci. 2015;14:1476–91. 25856800 10.1039/c4pp00495g PMC4520758

[143] Yano S, Hirohara S, Obata M, Hagiya Y, Ogura S-i, Ikeda A, et al. Current states and future views in photodynamic therapy. J Photochem Photobiol C Photochem Rev. 2011;12:46–67.

[144] Bugaj AM. Vascular targeted photochemotherapy using padoporfin and padeliporfin as a method of the focal treatment of localised prostate cancer-clinician’s insight. World J Methodol. 2016;6:65. 27019798 10.5662/wjm.v6.i1.65 PMC4804253

[145] Baskaran R, Lee J, Yang S-G. Clinical development of photodynamic agents and therapeutic applications. Biomater Res. 2018;22:25. 30275968 10.1186/s40824-018-0140-z PMC6158913

[146] Zafar I, Arfan M, Nasir R, Shaikh A. Aluminum phthalocyanine derivatives: potential in antimicrobial PDT and photodiagnosis. Austin Biomolecules Open Access. 2016;1:1–7.

[147] Kollár J. Syntéza anionických derivátů ftalocyaninů jako potenciálních fotodynamicky aktivních látek. 2020.

[148] Liu W-T, Wang H-T, Yeh Y-H, Wong T-W. An update on recent advances of photodynamic therapy for primary cutaneous lymphomas. Pharmaceutics. 2023;15:1328. 37242570 10.3390/pharmaceutics15051328 PMC10223676

[149] Lee E-H, Lim S-J, Lee M-K. Chitosan-coated liposomes to stabilize and enhance transdermal delivery of indocyanine green for photodynamic therapy of melanoma. Carbohydr Polym. 2019;224:115143. 31472877 10.1016/j.carbpol.2019.115143

[150] Zhang L, Wang K, Huang Y, Zhang H, Zhou L, Li A, et al. Photosensitizer-induced HPV16 E7 nanovaccines for cervical cancer immunotherapy. Biomaterials. 2022;282:121411. 35189461 10.1016/j.biomaterials.2022.121411

[151] Hu Y, Wang R, Zhou Y, Yu N, Chen Z, Gao D, et al. Targeted dual-mode imaging and phototherapy of tumors using ICG-loaded multifunctional MWCNTs as a versatile platform. J Mater Chem B. 2018;6:6122–32. 32254822 10.1039/c8tb01870g

[152] Damke GMZF, Damke E, de Souza Bonfim-Mendonca P, Ratti BA, de Freitas Meirelles LE, da Silva VRS, et al. Selective photodynamic effects on cervical cancer cells provided by P123 Pluronic®-based nanoparticles modulating hypericin delivery. Life Sci. 2020;255:117858. 32497635 10.1016/j.lfs.2020.117858

[153] Muhanna N, Chan HH, Townson JL, Jin CS, Ding L, Valic MS, et al. Photodynamic therapy enables tumor-specific ablation in preclinical models of thyroid cancer. Endocr Relat Cancer. 2020;27:41–53. 31751308 10.1530/ERC-19-0258

[154] Hsu C-W, Cheng N-C, Liao M-Y, Cheng T-Y, Chiu Y-C. Development of folic acid-conjugated and methylene blue-adsorbed Au@ TNA nanoparticles for enhanced photodynamic therapy of bladder cancer cells. Nanomaterials. 2020;10:1351. 32664275 10.3390/nano10071351 PMC7407911

[155] Lin T-Y, Li Y, Liu Q, Chen J-L, Zhang H, Lac D, et al. Novel theranostic nanoporphyrins for photodynamic diagnosis and trimodal therapy for bladder cancer. Biomaterials. 2016;104:339–51. 27479049 10.1016/j.biomaterials.2016.07.026 PMC5412594

[156] Li G, Yuan S, Deng D, Ou T, Li Y, Sun R, et al. Fluorinated polyethylenimine to enable transmucosal delivery of photosensitizer‐conjugated catalase for photodynamic therapy of orthotopic bladder tumors postintravesical instillation. Adv Funct Mater. 2019;29:1901932.

[157] Ding F, Li H-J, Wang J-X, Tao W, Zhu Y-H, Yu Y, et al. Chlorin e6-encapsulated polyphosphoester based nanocarriers with viscous flow core for effective treatment of pancreatic cancer. ACS Appl Mater Interfaces. 2015;7:18856–65. 26267601 10.1021/acsami.5b05724

[158] Hafiz SS, Xavierselvan M, Gokalp S, Labadini D, Barros S, Duong J, et al. Eutectic gallium–indium nanoparticles for photodynamic therapy of pancreatic cancer. ACS Appl Nano Mater. 2022;5:6125–39. 35655927 10.1021/acsanm.1c04353 PMC9150699

[159] Er Ö, Colak SG, Ocakoglu K, Ince M, Bresolí-Obach R, Mora M, et al. Selective photokilling of human pancreatic cancer cells using cetuximab-targeted mesoporous silica nanoparticles for delivery of zinc phthalocyanine. Molecules. 2018;23:2749. 30355983 10.3390/molecules23112749 PMC6278564

[160] Chen Z-A, Kuthati Y, Kankala RK, Chang Y-C, Liu C-L, Weng C-F, et al. Encapsulation of palladium porphyrin photosensitizer in layered metal oxide nanoparticles for photodynamic therapy against skin melanoma. Sci Technol Adv Mater. 2015;16:054205. 27877834 10.1088/1468-6996/16/5/054205 PMC5070020

[161] Mello VC, Araújo VHS, de Paiva KLR, Simões MM, Marques DC, da Silva Costa NR, et al. Development of new natural lipid-based nanoparticles loaded with aluminum-phthalocyanine for photodynamic therapy against melanoma. Nanomaterials. 2022;12:3547. 36296737 10.3390/nano12203547 PMC9609910

[162] da Silva DB, da Silva CL, Davanzo NN, da Silva Souza R, Correa RJ, Tedesco AC, et al. Protoporphyrin IX (PpIX) loaded PLGA nanoparticles for topical photodynamic therapy of melanoma cells. Photodiagnosis Photodyn Ther. 2021;35:102317. 33940210 10.1016/j.pdpdt.2021.102317

[163] Jardón-Guadarrama G, Manríquez-Ramírez ME, Rodríguez-Pérez CE.Díaz-Ruiz A, de los Ángeles Martínez-Cárdenas M, Mata-Bermudez A, et al. TiO2-ZnPc nanoparticles functionalized with folic acid as a target photosensitizer for photodynamic therapy against glioblastoma cells. J Mater Sci Mater Med. 2024;35:1–13. 10.1007/s10856-024-06823-w PMC11341649 39172269

[164] Kang RH, Kim Y, Um HJ, Kim J, Bang E-K, Yeo SG, et al. Glioblastoma homing photodynamic therapy based on multifunctionalized porous silicon nanoparticles. ACS Appl Nano Mater. 2022;5:5387–97.

[165] Wang X, Tian Y, Liao X, Tang Y, Ni Q, Sun J, et al. Enhancing selective photosensitizer accumulation and oxygen supply for high-efficacy photodynamic therapy toward glioma by 5-aminolevulinic acid loaded nanoplatform. J Colloid Interface Sci. 2020;565:483–93. 31982715 10.1016/j.jcis.2020.01.020

[166] Wang X, Li S, Liu H. Co-delivery of chitosan nanoparticles of 5-aminolevulinic acid and shGBAS for improving photodynamic therapy efficacy in oral squamous cell carcinomas. Photodiagnosis Photodyn Ther. 2021;34:102218. 33592329 10.1016/j.pdpdt.2021.102218

[167] Xu Y, Yang L, Wang C, Sun W, Zheng Y, Ou B, et al. Ferroptosis boosted oral cancer photodynamic therapy by carrier-free Sorafenib-Ce6 self-assembly nanoparticles. J Control Release. 2024;366:798–811. 38184236 10.1016/j.jconrel.2023.12.056

[168] Ren G, Wang Z, Tian Y, Li J, Ma Y, Zhou L, et al. Targeted chemo-photodynamic therapy toward esophageal cancer by GSH-sensitive theranostic nanoplatform. Biomed Pharmacother. 2022;153:113506. 36076595 10.1016/j.biopha.2022.113506

[169] Liu J, Gao J, Zhang A, Guo Y, Fan S, He Y, et al. Carbon nanocage-based nanozyme as an endogenous H 2 O 2-activated oxygenerator for real-time bimodal imaging and enhanced phototherapy of esophageal cancer. Nanoscale. 2020;12:21674–86. 33099588 10.1039/d0nr05945e

